# Comparative health systems analysis of differences in the catastrophic health expenditure associated with non-communicable vs communicable diseases among adults in six countries

**DOI:** 10.1093/heapol/czac053

**Published:** 2022-07-12

**Authors:** Annie Haakenstad, Matthew Coates, Gene Bukhman, Margaret McConnell, Stéphane Verguet

**Affiliations:** Department of Global Health and Population, Harvard T.H. Chan School of Public Health, 677 Huntington Avenue, Boston, MA 02115, USA; Institute for Health Metrics and Evaluation, University of Washington, 3980 15th Avenue NE, Seattle, WA 98195, USA; Center for Integration Science, Division of Global Health Equity, and Division of Cardiovascular Medicine, Brigham and Women’s Hospital, 75 Francis Street, Boston, MA 02115, USA; Center for Integration Science, Division of Global Health Equity, and Division of Cardiovascular Medicine, Brigham and Women’s Hospital, 75 Francis Street, Boston, MA 02115, USA; Program in Global Noncommunicable Disease and Social Change, Harvard Medical School, 641 Huntington Avenue, Boston, MA 02115, USA; NCD Synergies Project, Partners In Health, 800 Boylston Street, Suite 300, Boston, MA 02199, USA; Department of Global Health and Population, Harvard T.H. Chan School of Public Health, 677 Huntington Avenue, Boston, MA 02115, USA; Department of Global Health and Population, Harvard T.H. Chan School of Public Health, 677 Huntington Avenue, Boston, MA 02115, USA

**Keywords:** Costs, health systems research, health financing, financial risk protection, catastrophic health expenditure

## Abstract

The growing burden of non-communicable diseases (NCDs) in low- and middle-income countries may have implications for health system performance in the area of financial risk protection, as measured by catastrophic health expenditure (CHE). We compare NCD CHE to the CHE cases caused by communicable diseases (CDs) across health systems to examine whether: (1) disease burden and CHE are linked, (2) NCD CHE disproportionately affects wealthier households and (3) whether the drivers of NCD CHE differ from the drivers of CD CHE. We used the Study on Global Aging and Adult Health survey, which captured nationally representative samples of 44 089 adults in China, Ghana, India, Mexico, Russia and South Africa. Using two-part regression and random forests, we estimated out-of-pocket spending and CHE by disease area. We compare the NCD share of CHE to the NCD share of disability-adjusted life years (DALYs) or years of life lost to disability and death. We tested for differences between NCDs and CDs in the out-of-pocket costs per visit and the number of visits occurring before spending crosses the CHE threshold. NCD CHE increased with the NCD share of DALYs except in South Africa, where NCDs caused more than 50% of CHE cases but only 30% of DALYs. A larger share of households incurred CHE due to NCDs in the lowest than the highest wealth quintile. NCD CHE cases were more likely to be caused by five or more health care visits relative to communicable disease CHE cases in Ghana (*P* = 0.003), India (*P* = 0.004) and China (*P* = 0.093). Health system attributes play a key mediating factor in how disease burden translates into CHE by disease. Health systems must target the specific characteristics of CHE by disease area to bolster financial risk protection as the epidemiological transition proceeds.

Key messages

- We compared health system performance for financial risk protection for non-communicable disease (NCD) vs communicable disease care in China, Ghana, India, Mexico, Russia and South Africa.
- The NCD share of catastrophic health expenditure (CHE) generally increased with the NCD share of disability-adjusted life years (DALYs) in the six countries with the exception of South Africa where NCDs were a much higher share of CHE than disease burden.
- Compared with the wealthiest households, a larger share of the poorest households incurred CHE due to NCDs, highlighting that financial risk protection for NCDs could be connected to broader poverty alleviation goals.
- As compared with communicable disease CHE cases, NCD CHE cases were more likely to be the result of spending from many visits rather than a single, large spending shock. Households thus face different types of welfare loss depending on the disease driving CHE—the steady, cumulative nature of NCD costs may be more predictable and less likely to require displacement of spending on essential goods and services. As the epidemiological transition proceeds, financial risk protection policies will need to address CHE that culminate over many healthcare encounters, as seen for NCDs, for example by lowering the cost of NCD medicines and other out-of-pocket costs that are small on a one-time basis but add up with frequent use.

## Introduction

A key measure of health system performance is financial risk protection, typically measured by catastrophic health expenditure (CHE) or when out-of-pocket (OOP) health expenditure surpasses a given threshold of consumption expenditure or income (Roberts *et al.*, 2003; World Health Organisation (WHO) 2010). Based on a 40% capacity-to-pay threshold, CHE affected an estimated 210 million people worldwide in 2010 Wagstaff *et al.* (2017). Better information on how to reduce the substantial number of households affected by financial hardship due to health care costs is critical to improving health system performance and supporting countries in their pursuit of universal health coverage United Nations (UN) (2018).

The challenge of reducing CHE is complicated by the need for health systems to confront the healthcare needs of rapidly ageing populations and the growing burden of non-communicable diseases (NCDs) (Binagwaho *et al.*, 2014; Vollset *et al.*, 2020; Vos *et al.*, 2020). There is reason to believe that NCD care, particularly for cardiovascular disease and cancer, is more expensive than care for maternal and child health and infectious diseases (van Mourik *et al.*, 2010; Khatib *et al.*, 2016). The lack of development assistance for health (DAH) for NCDs and growing evidence that government health spending in low- and middle-income countries does not focus on NCDs, suggest the costs of care are more likely to be passed on to patients Institute for Health Metrics and Evaluation (IHME) (2020). Much of the international and government investments focus instead on communicable diseases (CDs), potentially making healthcare for CDs more likely to be subsidized. The low level of pooled funding and potentially higher OOP costs could mean that increases in the burden of NCDs are likely to increase CHE—as long as care is accessible. A recent comparison of a subset of NCD vs CD care showed that coverage rates are lower for NCDs than CDs except at high levels of development Murray *et al.* (2020). Within-country inequities in access to NCD care, including cost barriers to private sector NCD care, could mean low-income NCD patients do not have access to the care they need and thus do not incur CHE. The connection between the rise of NCDs and CHE rates thus depends on the burden of disease, but also key health system features such as financing, access, inequities and the role of the private sector in healthcare delivery.

There is limited cross-country evidence about the contribution of NCDs to CHE and how the mechanisms driving NCD CHE might differ from other disease areas and across health systems. Comparing financial risk protection by disease area across countries permits consideration of which factors are common across contexts and which factors are due to particularities of health systems, including their financing and access policies. A number of recent single-country studies have assessed CHE by disease area (Mahal *et al.*, 2010; Engelgau *et al.*, 2011; 2012; Smith-Spangler *et al.*, 2012; Verguet *et al.*, 2016; Selvaraj *et al.*, 2018; Kastor and Mohanty, 2018; Sangar *et al.*, 2019), but such analyses have not yet been conducted in a large number of countries, limiting any capacity to make conclusions about systematic differences in CHE by disease area across health systems. Many estimates of CHE due to NCDs have focused on a subset of NCD conditions (e.g. studies focused solely on heart disease or cancer or diabetes) (Smith-Spangler *et al.*, 2012; Jan *et al.*, 2018; Haakenstad *et al.*, 2019), rather than the NCD group on the whole, limiting generalizability to the epidemiologic transition overall. Furthermore, while meta-analysis extending results from a small set of countries to the world has been conducted Essue *et al.* (2018), a systematic review concluded the lack of representative data in existing studies prohibits such meta-analysis Kankeu *et al*. (2013). Finally, while many studies have associated CHE with a range of individual, household and health system factors (Sharma *et al.*, 2018; Njagi *et al.*, 2018; Fernandes Antunes *et al.*, 2018; Wang *et al.*, 2018), none have investigated the characteristics of disease-specific CHE and how it differs across health systems. Given the differences in access, financing and epidemiology that characterize CDs vs NCDs, we focus on comparing these two distinct disease areas and the financial hardship they respectively cause.

In this study, we use comparative health systems analysis to assess differences in NCDs vs CDs in CHE rates, equity in CHE and drivers of CHE. We apply random forests and two-part regression methods to survey data from the World Health Organisation (WHO) Study on Global Aging and Adult Health (SAGE). We had three aims. First, we compared CHE for NCDs in six major low- and middle-income countries (China, Ghana, India, Mexico, Russia and South Africa) against their burden of disease, to assess whether NCD CHE increases as the NCD burden grows. Second, we examine the equity implications for CHE due to NCDs, to assess whether NCD CHE is more likely among individuals in wealthier households or individuals in poorer households. Finally, we investigate which potential drivers of OOP costs in these countries are more prominent for NCDs vs CDs. We focus on differences in the use of private sector vs public sector care, based on the concern that NCD care may be more accessible in private sector facilities catering to wealthier individuals that were more likely to have NCDs historically. We also assess whether costs or utilization intensity are bigger drivers of NCD CHE vs CD CHE, since the recommended policy would differ depending on whether CHE results from a one-time shock, which could be more common for the acute, one-time encounters associated with CDs or, alternatively, whether CHE results from the culmination of spending over many visits, which is more likely for NCDs since these diseases tend to be more long-lasting and require regular follow-up. Overall, with analysis focused on understanding the connection between CHE and the rising NCD burden, our study aims to support the identification of potential interventions to improve financial risk protection and more broadly improve health system performance.

## Materials and methods

### Data

The SAGE surveys were conducted from 2007–2010 in China, Ghana, India, Mexico, Russia and South Africa and employed a multistage cluster sampling design to select participating households. The survey methodology and sampling have been described in depth elsewhere Kowal *et al.* (2012), and we briefly summarize them here. The SAGE surveys captured a nationally representative sample of 44 089 adults older than 18 years of age, with sample sizes ranging from 15 009 respondents in China to 2742 in Mexico, with an oversampling of adults aged 50 years and older. We used the survey weights provided by the SAGE to adjust all quantities of interest to be nationally representative of adults. Paper-based questionnaires were applied through face-to-face interviews in Ghana, India, Russia and South Africa. In Mexico and half of the interviews in China, face-to-face interviews were computer-assisted. Consent was obtained through standardized procedures detailed in the study protocol WHO (2006). Households were classified as either 18–49‐years‐aged households or 50+‐years‐aged households. Individual and household questionnaires were applied. For the individual questionnaire, one individual was selected from 18–49 households; in 50+ aged households, all individuals older than 50 years were selected. The individual questionnaire focused on health and well-being, health care utilization and health spending per visit, among other questions. One household questionnaire was completed per household. The respondent for the household questionnaire was not necessarily the respondent in the individual survey. The household questionnaire captured the household roster, wealth, spending, income and other sociodemographic characteristics. We obtained approval to use the SAGE surveys from the WHO Multi-Country Studies Data Archive, per WHO guidelines WHO (2019).

### Identifying the cause of visit by disease area

SAGE respondents were asked to identify the reason for seeking care for their three most recent inpatient stays and three most recent outpatient visits. Respondents selected among 18 distinct health reasons for these visits. Informed by the groupings developed by the Global Burden of Disease (GBD) study IHME (2018), we categorized these responses into seven groups: NCDs, CDs (which includes maternal and child health, in line with GBD groupings), injuries, pain, surgery, other and unidentified.

Respondents did not report the reason for seeking care for the fourth most recent visit and other prior encounters but did report the total number of inpatient and outpatient visits in the past year. We imputed the missing cause of visits using random forests. Random forests were selected because it outperformed other methods based on out-of-sample validation Caruana and Niculescu-Mizil (2006), and predictive accuracy was high (appendix p. 9).

### Computing OOP expenditure by disease area

SAGE respondents also indicated how much they spent OOP on their most recent inpatient and outpatient visits, respectively. The costs of other visits were not captured. OOP costs are defined as spending at the time of visit, including on medicine, consultation fees, tests and other medical costs. Informal payments to medical providers were not included. We are unable to further distinguish the type of care used—including whether a visit was for more advanced care (e.g. invasive procedure) or a basic consultation to manage a chronic condition. These features of a visit could have implications for OOP costs that we are unable to address. We used reported OOP spending from the most recent visit to model costs for prior visits. We converted OOP spending to 2017 international dollars (adjusted for purchasing power parity) and applied a two-part logit-log-link generalized linear regression model to predict OOP spending per visit by cause of visit (appendix p. 12–15). Covariate selection was based on out-of-sample root-mean squared error.

To calculate annual OOP spending by disease area for each respondent, we multiplied the cause of visit by the OOP cost for each individual, disease and visit. For visits 1–3, we used the reported cause. For visit 1, we used the reported OOP cost. For all other visits, we used the predicted cause of the visit and the visit’s predicted OOP spending. OOP spending was then summed across all visits to generate annual OOP spending by disease for each respondent.

### Estimating CHE

CHE was based on whether OOP health spending exceeded 40% of the capacity-to-pay (Xu *et al.*, 2003; Wagstaff *et al.* 2017). Capacity-to-pay was calculated as the difference between annual household expenditure and the mean of the 45th to 55th percentile of food consumption expenditure, scaled by household size. The OOP costs used in the CHE calculation capture total spending for the household for the year. They are the total of all visits reported by respondents (total outpatient and inpatient visits) multiplied by the costs for each visit (the observed most recent cost and imputed costs for visits beyond the most recent).

In more than 70% of CHE cases, all OOP spending was associated with one spending category and we assigned CHE cases to that category. For people with a mix of different spending categories, each CHE case was assigned to a disease-specific spending category (NCDs, CDs or injuries) if OOP spending on that disease comprised more than 75% of disease-specific OOP spending. All other CHE cases were considered ‘unallocable’. Uncertainty intervals (UIs) were generated using 1000 draws with a non-parametric bootstrap, resampled by strata to incorporate SAGE’s complex survey design Kovar *et al.* (1988).

We compare the share of CHE due to NCDs to the share of disability-adjusted life years (DALYs) due to NCDs among adults aged 20 years and older as estimated by the GBD Study 2019 GBD 2019 Diseases and Injuries Collaborators (2020). We also assess how rates of CHE and the share of CHE due to NCDs differ across wealth quintiles using wealth quintiles provided by the SAGE survey team.

### Assessing drivers of CHE: utilization and OOP spending

We examined patterns of disease-specific utilization and OOP spending for the most recent inpatient and outpatient visit, using the non-modelled information available from the SAGE survey. We used ordinary least squares (OLS) regression models to examine the variation in OOP spending per visit and private facility attendance. OLS regressions were implemented separately by country and for inpatient and outpatient visits with the following covariates: an indicator for each disease category (NCDs, CDs, injuries and other), age, an indicator for urban residence, educational attainment, sex and wealth quintile. Standard errors were clustered by primary sampling unit.

Finally, we examined how CHE cases differed according to the number of visits that occurred for OOP spending to exceed the 40% capacity-to-pay threshold, which we called the number of ‘visits-to-CHE’. The visits-to-CHE number was based on ranking visit OOP spending from the highest to the lowest spending amount and calculating culminative OOP spending for each additional visit, starting with the most expensive visit. To test whether these characteristics were distinct by disease area, we regressed the probability that: (1) CHE was caused by one visit and (2) the probability that CHE was caused by five or more visits on: an indicator for the disease type causing CHE, age, an indicator for urban residence, educational attainment, sex, wealth quintile and restricting to CHE cases only. Regressions were conducted separately for each country and standard errors were clustered by the primary sampling unit. Note that a key assumption in this approach is that the OOP costs for one visit are independent of the costs of other visits for a given individual and household. This assumption was required because we do not have OOP costs for sequential visits from which to conduct inference. This assumption entails ignoring the possibility that insurance programmes or catastrophic health funds kick in once OOP costs exceed a certain amount. This would bias our results if the programmes came into effect below the CHE threshold of 40% capacity-to-pay we used. We do not believe that is the case in any of the countries studied.

More details on all aspects of the modelling processes are available in the supplementary appendix. All analyses were conducted with R statistical software (version R 3.4.0) and STATA software (version 14.0).

## Results

The characteristics of the respondents in the SAGE surveys varied from country to country (Table 1). The mean age ranged from 41 to 48 years, and the share of individuals living in rural areas ranged from 22% (Mexico) to 69% (India). Outpatient care ranged from 1.4 (Ghana) to 1.8 (Mexico) visits per person per year. Annual hospitalizations per person ranged from 0.04 in Mexico to 0.15 in Russia.

**Table 1.** Descriptive statistics from the Study on Global Aging and Adult Health (SAGE) surveys

Country	Response rates	Number of individuals surveyed	Mean age (years)	Share female (%)	Share rural (%)	Average number of outpatient visits (per person per year)	Average number of inpatient visits (per person per year)
China	93%	15 009	48 (0.2)	50.2 (1.0)	52.5 (0.1)	1.8 (0.10)	0.11 (0.01)
India	68%	12 196	41 (0.3)	48.6 (5.8)	68.8 (2.4)	1.6 (0.11)	0.09 (0.01)
Mexico	53%	2741	43 (0.9)	52.1 (3.8)	22.3 (3.3)	1.6 (0.38)	0.04 (0.02)
Russia	56%	4355	47 (1.2)	54.9 (3.8)	25.9 (5.2)	1.7 (0.16)	0.15 (0.02)
South Africa	75%	4223	42 (0.8)	52.5 (3.7)	30.8 (2.9)	1.5 (0.08)	0.12 (0.04)
Ghana	81%	5565	45 (0.3)	50.4 (1.6)	54.0 (1.3)	1.4 (0.08)	0.10 (0.01)

Note: Standard errors are indicated in parentheses. SAGE survey weights and complex survey design are applied in the computation of all metrics. Response rates for all individuals according to (Kowal *et al.* 2012; Smith-Spangler *et al.*, 2012).

**Figure 1.**
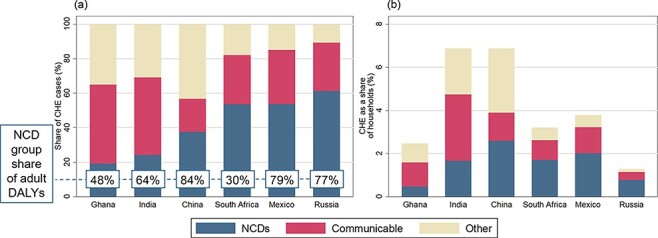
Distribution of catastrophic health expenditure (CHE) by disease area in China, Ghana, India, Mexico, Russia, and South Africa

Note: Disability-adjusted life years (DALYs) due to non-communicable diseases (NCDs) as a share of total DALYs for adults aged 20 and older from the Global Burden of Disease (GBD) study 2019.

The distribution of CHE cases by disease area is presented as a share of all CHE cases, with the NCD share of adult DALYS depicted in the box on each country’s bar (Figure 1a). Figure 1b depicts CHE cases by disease area as a share of all households. NCD-induced CHE comprised at least 20% of all CHE adult cases in all countries and was at least 50% of disease-specific CHE in Mexico, Russia and South Africa. China had the largest share of households affected by NCDs (2.6%, 95% UI: 2.3–2.9%), and NCDs were the biggest proportion of CHE cases in Russia (62.5%, 44.9–83.0%). CD-induced CHE was largest in India as a share of households (3.1%, 2.7–3.5%), and, as a share of all CHE cases, was largest in Ghana (45.0%, 32.2–57.1%) and India (44.7%, 40.7–48.5%).

As the NCD share of DALYs rose so generally did the NCD share of CHE (Figure 1a). Mexico and Russia had the second- and third-highest NCD shares of both DALYs and CHE, while India and Ghana had the lowest NCD shares. South Africa was distinct in that, although NCDs constituted more than 50% of all CHE cases, they made up 30% of DALYs. In China, NCDs comprised 84% of DALYs, substantially more than the estimated NCD share of CHE (38%, 95% UI: 34–42%).

Because fewer than 5000 individuals were sampled in Mexico, Russia and South Africa and CHE rates overall are low in these countries, there were less than 25 CD CHE cases in our sample for those three countries. This prohibited inference regarding the differences in drivers of CHE by disease in these countries. Results for all countries are available in the appendix but we focus the remainder of the analysis on China, Ghana and India.

**Figure 2.**
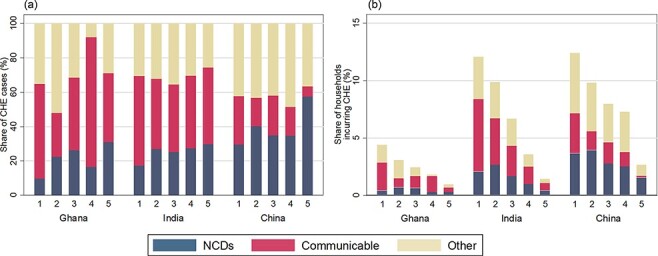
Distribution of catastrophic health expenditure (CHE) by disease area in China, Ghana, and India by wealth quintile

Note: Numbers 1–5 indicate wealth quintiles 1–5 where 1 is the lowest and 5 is the highest.

Figure 2 depicts CHE broken down by cause and wealth quintile for China, Ghana and India (results for all countries in the appendix p. 18–21). In all three countries, as wealth quintile increases, a larger share of the CHE cases is attributed to NCDs (Figure 2a). However, because CHE rates are substantially higher in the lowest wealth quintile than the highest wealth quintile, the share of households affected by NCD CHE is highest in the lowest wealth quintile in all three countries (Figure 2b).

**Figure 3.**
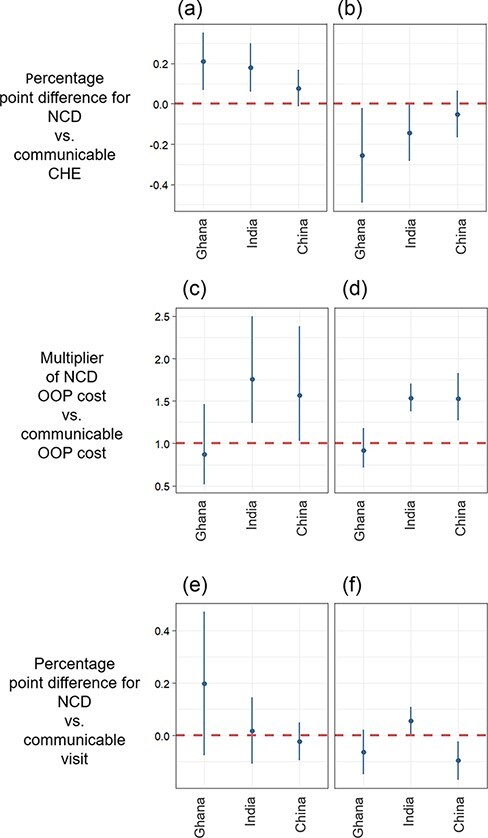
Testing NCD vs CD differences in the number of visits that occurred to push out-of-pocket OOP health spending over the catastrophic health expenditure (CHE) threshold, OOP spending and utilization in China, Ghana and India. (a) Five or more visits to CHE, (b) One visit to CHE, (c) Inpatient OOP, (d) Outpatient OOP, (e) Inpatient Private Facility and (f) Outpatient Private Facility.

Note: Points capture coefficient estimates and bars capture coefficient uncertainty intervals from an ordinary least squares regression of the dependent variable labelled in each figure’s title. The number of visits to CHE captures the cumulative number of visits that occurred before the CHE took place. Visits were ranked from the most expensive to the least expensive. OOP spending was then calculated cumulatively, based on the rank-order of visits, and then compared with capacity-to-pay to assess how many visits occurred before spending was pushed above the 40% capacity-to-pay CHE threshold. ‘One visit to CHE’ is an indicator of whether or not one visit pushed OOP health spending over the CHE threshold. ‘Five or more visits to CHE’ is an indicator of whether or not five or more visits pushed OOP health spending over the CHE threshold. For other figures (c, e, f, g), the dependent variable is based on the most recent inpatient or outpatient visit and visits caused by CDs are the reference category. Other controls in the regression model included indicators for whether a visit was due to an injury or another cause, wealth quintile, educational attainment, age, sex and an indicator for urban residence. All regressions were run with survey weights and robust standard errors were clustered by primary sampling unit. Full regression results are available in Tables S10–S23 (Supplementary Appendix p. 22–35).

Figure 3 depicts regression coefficients and confidence intervals for indicators representing the difference in six outcomes for NCDs vs CDs, controlling for socioeconomic and health system characteristics. Results for all countries available in the appendix (p. 22–35). First, Figure 3a shows that NCD CHE was more likely to be the result of the culmination of spending over many visits: the probability that five or more visits were required for CHE to occur was higher for NCD CHE cases than CD CHE cases in Ghana (21.0 p.p., 6.7–35.2, *P* = 0.003), India (18.0 p.p., 6.0–29.9, *P* = 0.004) and China (7.8 p.p., −1.3–16.9, *P* = 0.093). NCD CHE cases were less likely to be caused by a single spending shock, as represented by the probability that one visit alone resulted in CHE, in Ghana (25.6 p.p., 2.2–48.9, *P* = 0.032) and India (14.4 p.p., 0.4–28.4, *P* = 0.044) (Figure 3b). Both inpatient and outpatient costs were approximately 1.6 times higher for NCDs than for CDs in China (*P* < 0.001 and *P* = 0.037) and India (*P* < 0.001 and *P* = 0.002) but did not differ significantly in Ghana (Figures 3c and d). The probability of using a private facility did not differ for inpatient care (Figure 3e). The probability of using a private facility for outpatient care, in contrast, was 5.6 percentage points (0.3 to 10.8) higher for NCDs than CDs in India (*P* = 0.037) and 9.5 percentage points (2.3 to 16.8) lower in China (*P* = 0.011) (Figure 3f).

## Discussion

This study quantified the distribution of CHE by disease area in six countries and showed that NCD CHE tends to increase with the NCD share of DALYs. Major exceptions, like South Africa and China, show that the health system plays a critical role in mediating the relationship between the burden of disease and CHE by disease area, however. NCD CHE rates were higher in the lowest wealth quintiles although the NCD CHE share of all CHE cases rose with wealth. As compared with CD CHE cases, NCD CHE cases were more likely to be the result of the culmination of spending over many visits rather than a one-time shock.

Contextualizing the results with disease burden facilitated comparison across countries and highlighted critical distinctions in health systems In Mexico and Russia, the NCD share of CHE and disease burden were higher than most countries. NCDs constituted the largest share of DALYs and CHE cases in these two countries GBD 2019 Diseases and Injuries Collaborators (2020). In South Africa, although NCDs constituted just 30% of DALYs, they made up more than 50% of all CHE cases. This divergence between the burden of disease and CHE is likely related to the segmentation of the South African health system: wealthier South Africans, who are impacted more by NCDs, typically turn to private health services where OOP payments can be substantially higher, while poor South Africans may have difficulty accessing care for NCDs Ele-Ojoe Ataguba and Akazilla (2010). The result in South Africa thus may be connected to inequities in South Africa’s health system. Furthermore, international donors and the government of South Africa invest heavily in addressing the human immunodeficiency virus, acquired immunodeficiency syndrome (HIV/AIDS) epidemic and making antiretrovirals and other care affordable, which may explain the smaller relative share of CD CHE Institute for Health Metrics and Evaluation (IHME) (2020). The sample size limited our ability to further analyse the drivers of CHE by disease area in Mexico, Russia and South Africa.

In China, the NCD share of DALYs was more than 50% higher than the NCD share of CHE. While we would not expect the shares to be perfectly aligned, we note that OOP expenditure on inpatient stays and outpatient care was also nearly 50% higher for NCDs than for CDs, suggesting that NCD care is more expensive but also that low NCD CHE rates may be related to foregone care due to costs rather than financial risk protection. This aligns with evidence that NCDs have not been highly prioritized by the government, translating into limited access to NCD prevention and treatment services for many low-income populations Tang *et al.* (2013). Treatment for communicable causes is, in contrast, more widely available, providing populations with more opportunities to incur CHE for those conditions. Addressing costs of NCD care as well as access to care in China is paramount for addressing the healthcare needs of the growing NCD burden. Finally, we note that a substantial share of CHE cases could not be tied to a disease area in China, limiting some of the inference possible for this country.

In India, NCDs caused 25% of all CHE cases and 64% of adult DALYs. Like China, OOP expenditure on inpatient stays and outpatient care were more than 50% higher for NCDs than for CDs, aligning with the hypothesis that costs of this type of care are higher. Outpatient care was more likely to be sought in the private sector for NCDs than CDs in India. Previous studies have shown that a substantial share of health care is sought in the private sector in India Kastor and Mohanty (2018). Furthermore, the Indian government has made major investments to improve access to public services for maternal care, in particular Lim *et al.*, 2010), with 75% of women benefiting from the Janani Suraksha Yojana (JSY) conditional cash transfer scheme for institutional deliveries Randive *et al.* (2013).

In Ghana, CHE and DALYs were lowest across the countries studied: NCDs made up nearly 20% of cases and 48% of DALYs. Utilization and OOP spending patterns were not distinct by cause in Ghana however in contrast to China and India. The National Health Insurance Scheme (NHIS) theoretically covers the bulk of the costs for both cause categories (CDs and NCDs), and while the programme has not been as pro-poor as originally intended Kotoh (2016), the lack of differentiation in OOP spending by cause could be a reflection of NHIS’s design.

The patterns in how CHE arose by wealth quintile and drivers by cause highlighted the potential for adopting targeted CHE reduction strategies by disease area. First, in all three countries examined, the lowest wealth quintile had a higher share of households affected by NCD CHE than the highest wealth quintile. This highlights that financial risk protection for NCDs would not benefit just the well-off, and could be connected to the broader poverty alleviation agenda. Second, in Ghana and India, CD CHE cases were more likely to be caused by a single, expensive visit. Large OOP spending shocks driven by unexpected health events are more difficult to anticipate and can jeopardize the ability of households to smooth consumption, displacing expenditure that would otherwise be used for essential goods and services such as housing, food and education costs Flores and O’Donnell (2016). These events could be addressed by strategies directly mitigating large OOP spending associated with CDs, like catastrophic health funds Lozano and Garrido (2019). Third, in all three countries, NCD CHE cases were more likely to be caused by the culmination of OOP spending over many visits, with the probability of CHE due to five or more visits significantly higher for NCD CHE than CD CHE. Such repetitive spending events may make it easier for households to plan for spending and smooth consumption, and thus may be better for household welfare. Although catastrophic funds could also be useful for people who incur NCD CHE, reducing these higher repetitive per visit costs, for instance by instituting subsidized drug prices (see examples for HIV/AIDS, Waning *et al*. (2009) malaria, AMFm Independent Evaluation Team (2012) and vaccines Nguyen *et al.* (2011)) or directly financing NCD services via government or donor funds, could substantially alleviate the financial burden of the growing NCD-affected population.

Overall, this multi-country analysis shed light on similarities and differences in CHE by disease area and how it arises, underscoring the critical role of health systems in intermediating the link between disease and financial hardship. While insurance programmes and other health system features have changed in many countries since these data were collected, our analysis has a number of general features that pertain to addressing financial risk protection today. First, we showed that the OOP costs for NCDs tended to be higher as compared with the OOP costs of CDs, but this is not universal—OOP costs for NCDs were higher in India and China but not in Ghana. Second, we showed that CHE by disease area is not directly aligned with disease burden. Insurance programme features, like the segmentation of insurance programmes in South Africa, are critical to the access and financial coverage of NCDs. Disease burden is not a proxy for rates of CHE by disease—CHE by disease area must be calculated itself to better understand which diseases are at the root of financial hardship. Third, our analysis showed that analysing CHE by the disease can be an important input to pursuing UHC in a cost-effective manner. It permits the identification of the disease areas most contributing to CHE and thereby lays the foundation for designing disease-specific policies to address CHE—the specific services, medications and diagnostics that need to be covered in health benefits packages to reduce CHE and advance UHC. Without disease-specific CHE, it is difficult to determine which benefits would most reduce financial hardship. Our analysis highlights that focusing on disease-specific CHE, rather than total CHE, is required to understand the drivers of poor financial risk protection and design effective policies. Finally, our analysis underscored that, because NCD CHE is less likely to arise from a one-time shock, it requires different policies than those most effective for CDs. Insurance programmes that do not cover outpatient care or medications, for example in India, are likely to be missing the OOP spending that culminates over many visits to CHE. Coverage of these types of small healthcare spending events should be considered as the burden of NCDs rises.

Our study has limitations related to data and methodology. First, only 18 broad response options were provided in the SAGE questionnaire, limiting our ability to fully allocate CHE cases across all disease areas. Our analysis focused only on disease burden and CHE caused by adult care, which could lead to an underestimation of the share of CHE associated with CD causes (e.g. childhood vaccine-preventable diseases) and CHE rates overall. Second, we assumed respondents correctly recalled the cause of visits and the number of visits in the last year. Evidence from a number of settings indicates that respondents underestimate their health care utilization Ansah and Powell-Jackson (2013). Respondents are unlikely to remember accurately all visits in the last year, which would deflate our estimates of utilization (and thus CHE), but they are also more likely to remember large health spending events, which would inflate our estimates of OOP spending and CHE Das *et al.* (2011). Third, measuring spending at the individual level was required to connect spending to a disease area and utilization, which may result in lower CHE estimates than if assessed at the household level. Fourth, we were unable to allocate 20% of CHE cases in the three poorest countries (China, India and Ghana). Lack of investment in proper diagnosis, poor communication with patients or lack of education about disease areas could explain why patients were unable to identify the visit cause. Fifth, we imputed both the cause of visit and OOP spending. The cause of visit models did perform very well out of the sample (appendix p.9) but relied on extending relationships among the three most recent visits to other prior visits. Modelling OOP spending captured the mean visit OOP spending while smoothing over stochastic noise; as a result, OOP spending may be overestimated for some visits and underestimated for others. Finally, we note that the data we used in our study date back more than a decade. This limits their relevance for particular policy reforms in the country studied, where changes to financial risk protection systems have occurred since the surveys were fielded. We believe our study nonetheless provides general insights into how CHE arises differently depending on the disease area and the implications of those differences for equity.

## Conclusion

Given the significant and rising share of the disease burden of NCDs in low- and middle-income countries and the potentially high OOP costs associated with NCD care, policymakers must think critically about strategies to curb the NCD CHE burden. Comparing the differences in NCD CHE and CD CHE across countries provided support for developing policies targeted to both the disease area and the health system. Our study showed how investigating CHE by cause can provide evidence for developing and enacting reforms that promote financial risk protection and improve health system performance overall.

## Supplementary Material

czac053_Supp

## Data Availability

The SAGE survey data are available from the WHO (https://www.who.int/data/data-collection-tools/study-on-global-ageing-and-adult-health) upon request.

## Reflexivity statement

The authors include two females and three males and span multiple levels of seniority. The first author has experience conducting fieldwork in India and Ghana. An additional three co-authors have extensive experience in other LMICs. Three of the five co-authors specialize in health economics, another co-author specializes in medicine and anthropology and another co-author specializes in epidemiology.
