# Supplementary material for: Comparative health systems analysis of differences in the catastrophic health expenditure associated with non-communicable vs communicable diseases among adults in six countries
Source: Health Policy Plan. 2022 Jul 12;37(9):1107–15. doi: 10.1093/heapol/czac053 (PMC9557357; doi:10.1093/heapol/czac053)
Supplement: czac053_Supp [file czac053_supp.zip › SAGE Appendix March 2022 updated.docx]

Supplementary appendix

Supplement to: Comparative health systems analysis of differences in the catastrophic health expenditure associated with non-communicable versus communicable diseases among adults in six countries.

| Table of contents | Page |
| --- | --- |
| Annex 1: Estimating cause-of-visit by disease area | 3 |
| Annex 2: Estimating per visit out-of-pocket expenditure by disease area | 11 |
| Annex 3: Estimating catastrophic health expenditure by disease area | 15 |
| Annex 4: Characterizing utilization intensity and spending patterns | 21 |
| References | 35 |

Annex 1: Estimating cause of visit by disease area

Estimating cause of visit by disease area first required grouping each of the 18 response options provided in the World Health Organization (WHO) Study on Global Aging and Adult Health (SAGE) into broad Global Burden of Disease (GBD) categories. The exact response options provided and the GBD cause category each response optioj was tagged to are listed in Table S1. The categories listed in Table S1 are used to model utilization and out-of-pocket (OOP) expenditure. In the final estimates, all those categories with “unallocable” (i.e. no disease area could be assigned) are presented as belonging to that category.

**Table S1: Cause categories and response options**

| **Cause category** | **Response options** |
| --- | --- |
| Non-communicable diseases | - diabetes or related complications - heart problems - high blood pressure/hypertension - stroke/sudden paralysis - depression or anxiety - cancer - problems with mouth, teeth or swallowing - chronic pain in joints/arthritis |
| Communicable, maternal, neonatal and nutritional diseases | - communicable disease (HIV, infections, malaria, tuberculosis) - maternal and perinatal conditions (pregnancy) - nutritional deficiencies - acute conditions (diarrhoea, fever, flu, headaches, cough, other) |
| Injuries | - injury (not occupation-related) |
| Pain  (Unallocable) | - generalized pain (stomach, muscle or other nonspecific pain) |
| Surgery  (Unallocable) | - surgery |
| Other  (Unallocable) | - occupation/work related condition/injury - sleep problems - problems with breathing |
| Unidentified  (Unallocable) | - other, specify - don’t know |

## Random Forests approach: prediction problem

The SAGE surveys captured the cause of visit for the most recent visit, the second most recent and the third most recent visit. However, individuals were also asked about the number of outpatient visits and the number of inpatient visits in the last 12 months. The purpose of the cause-of-visit models was to predict the cause of visits beyond the third most recent visit (i.e. fourth most recent visit and beyond).

A visual example is presented in Figure S1. In this example, a respondent reports having 5 outpatient visits in the past year. In the example, the individual indicates that visit 1 was for an NCD, visit 2 was for pain and visit 3 was for an NCD; we have no information about the cause of visit for visits 4 and 5.

**Figure S1: Visual representation of data and modeling exercise for assigning the cause of visit for visits beyond the third most recent visit.**

Total Outpatient Visits in the last year = 5

**?**

**NCD**

**Pain**

**?**

**NCD**

Visit 2

Visit 3

Visit 4

Visit 5

Visit 1

**Model used to predict cause of these visits**

**Model fit**

The cause-of-visit models took advantage of the within-person relationships among the cause of visits 1, 2, and 3. This relationship was harnessed to predict cause of visit for visits 4 and 5, in the example presented, and for the fourth most recent visit and other visits further back in time for all other SAGE respondents.

## Modeling utilization: random forests model[[1]](#footnote-1)

## Our main approach to the prediction problem was to deploy random forest models. Random forests combine decision trees and bootstrap to classify each observation into one of the disease categories presented in Table S1. Random forests were our preferred method for two reasons: a) they are well adapted to classification problems with more than two categories; and b) measured with out-of-sample prediction, they are one of the best-performing machine learning techniques.[[2]](#endnote-1)

Decision trees classify observations into different categories by splitting observations at a series of nodes, often presented in the form of a decision tree, similar to Figure S2. At each node, observations are separated into two categories based on the sorting that minimizes the share of observations misclassified by the split. The classification error rate is used to determine the best split at each node. The classification error rate is the share of observations in a given grouping that do not belong to the most common class.

Starting with all observations, the model produces successive divisions, separating observations into a series of splits. At each split, a prediction for each observation is produced, assigning each the most commonly occurring class in the branch. The number of splits or branches depends on the number of observations in the final node and in our case was set to a minimum of five.[[3]](#endnote-2)

**Figure S2: Visual representation of a decision tree implemented in random forests models**

N = 100

N=67

N =33

N=5

N=28

N= 33

N=34

Split that minimizes classification error

Terminal node

**Class 3**

**Class 4**

**Class 1**

**Class 2**

Final classification

Random forests were developed in part in response to the recognition that a single decision tree tends to overfit, producing poor out-of-sample predictions. Random forests minimize this variance in two ways. First, at each node, only a random sample of covariates is used. Using a sub-sample of covariates ensures that highly predictive variables do not overpower classification, which can lead to overfitting and classification error. Second, random forest methods combine many decisions trees (in our case 500) to make the final classification of each observation. In each terminal node, observations are classified based on the most common category in their final grouping. Across the more than 500 trees we estimate, the most common classification is used as the final prediction.

**Observation classified as because it is the most common classification.**

**1**

**2**

**4**

**1**

**Figure S3: Visual representation of random forests**

500 decision trees created and the most common classification leads to the final classification prediction

## Assessing performance

We assessed performance of the random forests model with out-of-sample prediction. To test performance in a way that simulates our prediction problem, we trained the model on visits 1-2 and tested how well the model predicted visit 3. We tested the predictive power of the models by generating predictions of the out-of-sample cause of visit and calculating how often that classification aligned with true cause category.

An extensive range of covariates – more than 50 – were tested in the different models. Streamlining the number of covariates improved our classification error and thus not all covariates were ultimately deployed. We decided on our ultimate covariates based on which one had the highest variable importance and produced the lowest classification error for visit 3 predictions. Variable importance represents the mean decrease in the share of variables misclassified – i.e. classified to a category other than the observed category – in each node. We eliminated the variables with the lowest variable importance until classification error was minimized.

In both the inpatient and outpatient random forests models, selected covariates included: wealth quintile, age, sex, country, educational attainment, annual household expenditure, and the health area that caused each respondent’s most recent prior visit (lag cause of visit). Because they improved out-of-sample prediction, additional covariates were included in the outpatient model including: visit number (i.e. third most recent visit, fourth most recent visit, etc.), body mass index (BMI), and whether the respondent lived in a rural or urban area.

The random forests models performed well as measured by out-of-sample prediction, correctly classifying between 76.0% of outpatient visits and 81.9% of inpatient visits (Table S2). Out-of-sample prediction was highest for communicable diseases (CDs) and NCDs. The chronicity of care-seeking is likely an important driver of the high predictive validity for NCDs. The more erratic nature of contracting infectious diseases, one aspect of the CD category, likely leads to reduced precision in this area. The random nature of injuries also likely drives the poor precision for that cause.

**Table S2: Precision of cause-of-visit random forests models**

|  | **Outpatient** | **Inpatient** |
| --- | --- | --- |
| CD | 70.3% | 69.6% |
| Injury | 63.5% | 66.7% |
| NCD | 85.0% | 82.4% |
| Other | 72.5% | 88.8% |
| Unidentified | 67.6% | 85.1% |
| Pain | 65.9% | 75.0% |
| Surgery | 69.1% | 100.0% |
| Overall | 76.0% | 81.9% |

*Note:* Precision is calculated as the number of true-positive classifications divided by the total number of positive classifications (true positives plus false positives). The model was run on visits 1 and 2 only and predictions produced for the third most recent visit. Precision based on the predictions for visit 3 and is averaged across all trees.

Figures S4 and S5 capture the “variable importance” of the covariates included in the two random forests models, which is represented by the Gini index of node impurity. The higher the mean decrease, the higher the predictive power of the variable. The figures underscore how, for both types of health care service, the cause of the most recent visit (*“Lag cause of visit”*) is the most predictive – more than twice any other variable. Note that the values are larger for the outpatient model than the inpatient model, which is related to the larger outpatient sample size and the mode nodes created in the random forests model for outpatient visits.

**Figure S4: Variable importance of covariates in the random forest outpatient model**

##
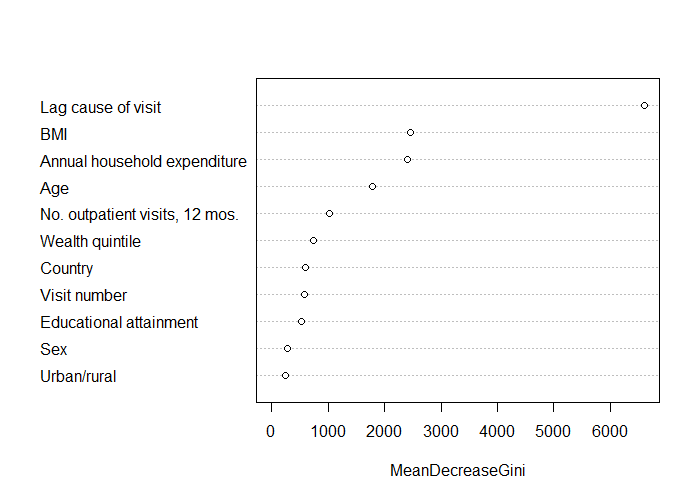

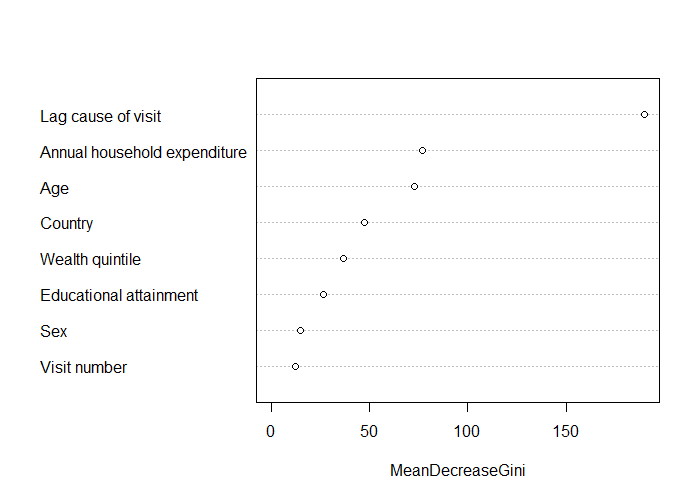


**Figure S5: Variable importance of covariates in the random forest inpatient model**

Mean decrease in the Gini index of node impurity

Mean decrease in the Gini index of node impurity

We generated the final predictions recursively. We first predicted visit 4 using the lag cause of visit 3. For visit 5, we used the lag cause of visit 4, and so on, until all visits reported by the respondents were tagged by cause. The SAGE survey captured the cause of visits throughout the year, and thus the model should be generalizable beyond the three most recent visits.

Annex 2: Estimating per visit out-of-pocket expenditure by disease area

Many respondents who used health services reported paying nothing. This high number of “zero spending” resulted in a zero-inflated lognormal distribution, which was difficult to model with available one-part models. We used a two-part regression model, an approach commonly used for fitting distributions of health expenditure.[[4]](#endnote-3),[[5]](#endnote-4) This two-part approach allowed us to model *whether* a health service user paid or not separately from *how much* health care users paid, given they expended some amount.

The two-part model developed by Belotti et al. (2012) was designed particularly for producing predictions with two-stage models, and we used the associated TPM package in STATA for estimating OOP expenditure for each cause, visit and individual.[[6]](#endnote-5) A key assumption of this approach is that the errors of the first and second stages are independent – the reasons for paying nothing are unrelated to how much is paid among those who pay. This assumption would not be valid, for example, if income determined whether or not a respondent paid nothing and how much was paid, but this type of system is not present in any of the six countries included in our study, to our knowledge.

The dependent variable is OOP spending on the most recent visit. This was converted to purchasing-power-parity-adjusted 2016 international dollars. For each reported inpatient or outpatient visit, we summed all sub-categories of reported expenditure to compute total cost per visit. Each respondent also reported the total spending on each visit. When there were discrepancies between these two values, we took the mean of the created and reported totals to generate our dependent variable.

We regress this OOP spending value on dummies for country, wealth quintile, and sex, because health system features as well as wealth and sex affect spending on health care. *Cause of visit 1* is a categorical variable capturing the reason for seeking care at the most recent visit, included in the model with a dummy representing each type of care and allowing us to estimate disease-specific OOP spending. *Total visits* represents the sum of all reported outpatient and inpatient visits in the last 12 months and is used as a proxy for severity of disease – assuming that respondents who used a lot of care tend to be sicker and thus have distinct per visit costs. Finally, we include a dummy for whether a respondent resided in an urban area, as the costs associated with care were likely to vary according to the availability and specialization of health providers and the distances traveled to access services. All variables were interacted to test the predictive power of additional dimensions of covariates. Covariates were selected based on the lowest RMSE calculated with 10-fold cross validation. Newton-Raphson iterations were limited to 200. Models that did not converge within 200 iterations were excluded.

**Table S3: Top five outpatient models and their average root mean squared error (RMSE)**

| Regression Number | Interactions | Average RMSE |
| --- | --- | --- |
| 10 | Wealth X Disease area; Country X Urban | 163.1 |
| 28 | Wealth X Disease area; Country X Visits | 163.2 |
| 13 | Wealth X Disease area; | 163.3 |
| 7 | Wealth X Disease area; Country X Female | 163.3 |
| 25 | Wealth X Disease area; Urban X Visits | 163.3 |

**Table S4: Top five inpatient models and their average root mean squared error (RMSE)**

| Regression Number | Interactions | Average RMSE |
| --- | --- | --- |
| 28 | Wealth X Disease area; Country X Visits | 3202.5 |
| 41 | Country X Wealth; Age X Disease area | 3205.4 |
| 25 | Wealth X Disease area; Urban X Visits | 3205.6 |
| 10 | Wealth X Disease area; Country X Urban | 3207.3 |
| 19 | Wealth X Disease area; Age X Urban | 3207 |

Table S5 displays the coefficients from the final two-part models selected. These models are run with the household survey weights for each country and are estimated with standard errors clustered at the household level.

**Table S5: Coefficients from two-stage OOP spending models**

| **(1) Outpatient OOP spending** | | **(2) Inpatient OOP spending** | |
| --- | --- | --- | --- |
| **LOGIT** |  | **LOGIT** |  |
| China (Reference) |  | China (Reference) |  |
| India | -0.300 | India | 0.276 |
| Mexico | -4.391*** | Mexico | -3.504*** |
| Russia | -4.014*** | Russia | -3.865*** |
| South Africa | -3.942*** | South Africa | -3.586*** |
| Ghana | -3.105*** | Ghana | 0.939 |
| Urban | -2.180*** | NCDs | -2.153* |
| India X Urban | 1.441* | Injury | -4.176* |
| Mexico X Urban | 3.076*** | Pain | 0.410 |
| Russia X Urban | 1.719** | Surgery | -2.250 |
| South Africa X Urban | 1.056 | Other | -0.756 |
| Ghana X Urban | 1.568* | Unidentified | -2.188 |
| NCDs | 0.374 | WQ2 | -2.056 |
| Injury | 0.620 | WQ3 | 1.405 |
| Pain | -0.565 | WQ4 | -0.747 |
| Surgery | 3.574*** | WQ5 | -2.864** |
| Other | 0.509 | NCDs X WQ2 | 2.243 |
| Unidentified | -0.904 | NCDs X WQ3 | -0.335 |
| WQ2 | -0.909 | NCDs X WQ4 | 1.296 |
| WQ3 | 0.272 | NCDs X WQ5 | 3.158* |
| WQ4 | 0.410 | Injury X WQ2 | 7.209** |
| WQ5 | 0.285 | Injury X WQ3 | -1.763 |
| NCDs X WQ2 | -0.239 | Injury X WQ4 | 3.190 |
| NCDs X WQ3 | -0.279 | Injury X WQ5 | 5.400** |
| NCDs X WQ4 | -1.573 | Pain X WQ2 | -0.351 |
| NCDs X WQ5 | -0.219 | Pain X WQ3 | -3.291 |
| Injury X WQ2 | -1.630 | Pain X WQ4 | 0.352 |
| Injury X WQ3 | -2.536 | Pain X WQ5 | -0.790 |
| Injury X WQ4 | -2.204* | Surgery X WQ2 | 2.674 |
| Injury X WQ5 | -1.323 | Surgery X WQ3 | 7.858** |
| Pain X WQ2 | 3.202* | Surgery X WQ4 | 1.705 |
| Pain X WQ3 | 1.024 | Surgery X WQ5 | 3.008* |
| Pain X WQ4 | 2.215 | Other X WQ2 | 1.318 |
| Pain X WQ5 | 0.599 | Other X WQ3 | -4.687 |
| Surgery X WQ2 | -3.158* | Other X WQ4 | 0.599 |
| Surgery X WQ3 | -2.552 | Other X WQ5 | 1.061 |
| Surgery X WQ4 | 0.266 | Unidentified X WQ2 | 1.784 |
| Surgery X WQ5 | -3.110** | Unidentified X WQ3 | -3.905 |
| Other X WQ2 | -1.002 | Unidentified X WQ4 | 0.742 |
| Other X WQ3 | -1.610 | Unidentified X WQ5 | 3.657* |
| Other X WQ4 | 0.379 | Female | 0.856 |
| Other X WQ5 | -0.443 | Total inpatient & outpatient visits (Visits) | -0.344* |
| Unidentified X WQ2 | 2.231** | Urban | -0.398 |
| Unidentified X WQ3 | 0.133 | Age | -0.0151 |
| Unidentified X WQ4 | -0.0786 | China X Visits | 0.340* |
| Unidentified X WQ5 | -1.455 | India X Visits | 0.586** |
| Female | -0.577* | Mexico X Visits | 0.427* |
| Total inpatient & outpatient visits (Visits) | 0.0278 | Russia X Visits | 0.423** |
| Age | -0.0297*** | South Africa X Visits | 0.141 |
| Constant | 7.523*** | Constant | 6.965*** |
| **GLM** |  | **GLM** |  |
| India | -0.300 | India | -0.726*** |
| Mexico | -4.391*** | Mexico | -0.176 |
| Russia | -4.014*** | Russia | -2.379*** |
| South Africa | -3.942*** | South Africa | -0.102 |
| Ghana | -3.105*** | Ghana | -2.493*** |
| Urban | -2.180*** | NCDs | 1.176*** |
| India X Urban | 1.441* | Injury | 2.173*** |
| Mexico X Urban | 3.076*** | Pain | 0.645* |
| Russia X Urban | 1.719** | Surgery | 1.197*** |
| South Africa X Urban | 1.056 | Other | 0.347 |
| Ghana X Urban | 1.568* | Unidentified | 0.905*** |
| NCDs | 0.374 | WQ2 | 0.663* |
| Injury | 0.620 | WQ3 | 0.890** |
| Pain | -0.565 | WQ4 | 0.893** |
| Surgery | 3.574*** | WQ5 | 0.973*** |
| Other | 0.509 | NCDs X WQ2 | 0.001 |
| Unidentified | -0.904 | NCDs X WQ3 | -0.804* |
| WQ2 | -0.909 | NCDs X WQ4 | -0.474 |
| WQ3 | 0.272 | NCDs X WQ5 | -0.019 |
| WQ4 | 0.410 | Injury X WQ2 | -0.671 |
| WQ5 | 0.285 | Injury X WQ3 | -1.347 |
| NCDs X WQ2 | -0.239 | Injury X WQ4 | -2.021** |
| NCDs X WQ3 | -0.279 | Injury X WQ5 | -0.740 |
| NCDs X WQ4 | -1.573 | Pain X WQ2 | -0.630 |
| NCDs X WQ5 | -0.219 | Pain X WQ3 | -0.903 |
| Injury X WQ2 | -1.630 | Pain X WQ4 | -1.666** |
| Injury X WQ3 | -2.536 | Pain X WQ5 | -0.081 |
| Injury X WQ4 | -2.204* | Surgery X WQ2 | -0.578 |
| Injury X WQ5 | -1.323 | Surgery X WQ3 | -0.147 |
| Pain X WQ2 | 3.202* | Surgery X WQ4 | -0.906* |
| Pain X WQ3 | 1.024 | Surgery X WQ5 | -0.399 |
| Pain X WQ4 | 2.215 | Other X WQ2 | -1.022 |
| Pain X WQ5 | 0.599 | Other X WQ3 | 0.519 |
| Surgery X WQ2 | -3.158* | Other X WQ4 | -1.812** |
| Surgery X WQ3 | -2.552 | Other X WQ5 | 0.224 |
| Surgery X WQ4 | 0.266 | Unidentified X WQ2 | -0.453 |
| Surgery X WQ5 | -3.110** | Unidentified X WQ3 | -0.504 |
| Other X WQ2 | -1.002 | Unidentified X WQ4 | -0.223 |
| Other X WQ3 | -1.610 | Unidentified X WQ5 | -0.685* |
| Other X WQ4 | 0.379 | Female | -0.158 |
| Other X WQ5 | -0.443 | Total inpatient & outpatient visits (Visits) | 0.073 |
| Unidentified X WQ2 | 2.231** | Urban | 0.167 |
| Unidentified X WQ3 | 0.133 | Age | -0.001 |
| Unidentified X WQ4 | -0.079 | China X Visits | -0.059 |
| Unidentified X WQ5 | -1.455 | India X Visits | -0.089 |
| Female | -0.577* | Mexico X Visits | -0.062 |
| Total inpatient & outpatient visits (Visits) | 0.028 | Russia X Visits | -0.037 |
| Age | -0.030*** | South Africa X Visits | -0.023 |
| Constant | 7.523*** | Constant | 6.195*** |
| N | 19654 | N | 3481 |
| * p<0.05, ** p<0.01, *** p < XXXX | | | |

Annex 3: Estimating catastrophic health expenditure by disease area

## OOP expenditure aggregation

To produce final annual OOP expenditure by cause for each respondent in the SAGE surveys, we multiply the observed and predicted values for both utilization (from the random forests model) and OOP (from the two-stage model) for each individual *i,* disease *d,* and visit *k*. For visits k=1-3, we use the observed cause of care and for visit k=1, we used the reported OOP value. For all other visits, we used the predicted cause and predicted OOP estimates from the models described in Annexes 1 and 2.

The spending and cause of visit were then summed across all visits to generate an annual OOP spending estimate, by disease *d*, for each respondent *i*, as shown in equation (1). Uncertainty intervals (UIs) were generated using n=500 draws with a non-parametric bootstrap, resampled at the stratum level to capture the variation introduced by the SAGE complex survey design.

where *OOPi,d,1* is observed and all other *OOPi,d,k* values are predicted and *Cause of Visiti,d,1-3*are observed and all other *Cause of Visiti,d,k>3* are predicted.

## Catastrophic health expenditure estimation

Catastrophic health expenditure (CHE) is defined as OOP health spending that surpasses 40% of capacity to pay, as shown in (2). Capacity to pay is defined as household spending minus the mean of the 45th to the 55th percentile of food expenditure in the population, adjusted for household size:

*

We use the adjustment for household size deployed in Xu et al. (2007), which uses decreasing returns to scale exponent of 1/2 as in equation (3).[[7]](#endnote-6) Food expenditure is first normalized with this adjustment:

## Tagging catastrophic health expenditure by disease area

We tagged each CHE case to a disease area, or alternatively to the “unallocable” category, based on the disease composition of OOP spending. More than 70% of people used care for the same broad disease area for all visits and thus all OOP spending was associated with that one disease area. This includes people who designated the cause of visit as “pain” or “other” for all visits and thus where CHE cases were tagged as “unallocable”.

The existence of these categories is due to the response options provided by the SAGE. If “pain” or “surgery” had not been a response option, it is possible that respondents would have selected a category more informative to a disease grouping. Furthermore, some response options were not designed to elicit a clear disease category. The response option “problems with breathing”, for instance, could be associated with a communicable disease (e.g. tuberculosis) or an NCD (e.g. chronic obstructive pulmonary disease). For this reason, some options could simply not be associated with any broad disease category and were allocated to “unallocable”.

For CHE cases that had a mix of OOP spending, we designated CHE by disease based on OOP spending associated with NCDs, injuries and CDs. CHE grouping was based on whether 75% of this disease-specific spending was associated with one of these three categories. Across all CHE cases, 12.6% were tagged in this way. (Relaxing the threshold to 50% would reallocate 14.5%; increasing the threshold to 99% would reallocate 11.3%.) CHE cases with less than 75% of all OOP spending going to one disease category were grouped into the unallocable category. CHE cases with only OOP spending on pain, surgery, other or unidentified were designated as unallocable.

**Table S6: Catastrophic health expenditure by disease area as a share of all surveyed individuals.**

|  | NCDs | CDs | Injuries | Unallocable |
| --- | --- | --- | --- | --- |
| China | 2.6% | 1.3% | 0.4% | 2.5% |
|  | (2.3 -2.9%) | (1.1-1.5%) | (0.3-0.6%) | (2.1-2.9%) |
| Ghana | 0.5% | 1.1% | <0.1% | 0.8% |
|  | (0.4%-0.6%) | (0.6%-1.5%) | (<0.1%-0.1%) | (0.5%-1.2%) |
| India | 1.7% | 3.1% | 0.2% | 1.9% |
|  | (1.4% - 2.0%) | (2.7%-3.5%) | (0.1%-0.3%) | (1.6%-2.2%) |
| Mexico | 2.0% | 1.2% | <0.1% | 0.6% |
|  | (0.7% - 3.1%) | (0.2%-2.1%) | (<0.1%-0.1%) | (0.2%-1.0%) |
| Russia | 0.8% | 0.4% | <0.1% | 0.1% |
|  | (0.5%-1.0%) | (<0.1%-0.8%) | (<0.1%-<0.1%) | (<0.1%-0.2%) |
| South Africa | 1.7% | 0.9% | <0.1% | 0.6% |
|  | (1.1%-2.3%) | (0.2%-1.6%) | (0.0%-<0.1%) | (0.3%-0.9%) |

Note: Uncertainty intervals in parentheses.

**Table S7: Catastrophic health expenditure (CHE) by disease are as a share of all CHE cases.**

|  | NCDs | CDs | Injuries | Unallocable |
| --- | --- | --- | --- | --- |
| China | 37.7% | 18.8% | 6.5% | 36.8% |
|  | (34.2-41.5%) | (16.1-21.5%) | (4.2-8.5%) | (32.9-40.7%) |
| Ghana | 19.5% | 45.0% | 1.1% | 33.9% |
|  | (14.9%-25.7%) | (32.2%-57.1%) | (0.3%-2.2%) | (22.1%-44.3%) |
| India | 24.3% | 44.7% | 3.4% | 27.6% |
|  | (20.8%-28.3%) | (40.7%-48.5%) | (1.8%-4.8%) | (24.1%-30.9%) |
| Mexico | 54.1% | 30.2% | 0.3% | 15.4% |
|  | (30.9%-76.7%) | (7.6%-56.1%) | (<0.1%-2.3%) | (7.1%-27.8%) |
| Russia | 62.5% | 26.2% | 0.5% | 10.7% |
|  | (44.9%-83.0%) | (.6%-44.4%) | (<0.1%-0.2%) | (3.7%-18.6%) |
| South Africa | 53.5% | 27.3% | 0.3% | 17.9% |
|  | (41.3%-69.8%) | (7.6%-41.5%) | (<0.1%-0.6%) | (9.3%-27.4%) |

Note: Uncertainty intervals in parentheses.

**Table S8: Catastrophic health expenditure by disease area as a share of all surveyed individuals by wealth quintile.**

| Country | Wealth Quintile | NCDs | CDs | Other |
| --- | --- | --- | --- | --- |
| China | 1 | 3.6% | 3.5% | 5.2% |
|  |  | (2.7%-4.5%) | (2.5%-4.3%) | (3.9%-6.4%) |
|  | 2 | 3.9% | 1.6% | 4.3% |
|  |  | (2.5%-5.4%) | (1.1%-2.1%) | (3.1%-5.4%) |
|  | 3 | 2.8% | 1.8% | 3.3% |
|  |  | (2.1%-3.7%) | (0.7%-2.6%) | (2.2%-4.2%) |
|  | 4 | 2.5% | 1.2% | 3.6% |
|  |  | (1.8%-3.1%) | (0.6%-1.7%) | (2.2%-4.7%) |
|  | 5 | 1.5% | 0.2% | 0.9% |
|  |  | (0.9%-2.1%) | (0.1%-0.2%) | (0.4%-1.4%) |
| Ghana | 1 | 0.5% | 2.5% | 1.6% |
|  |  | (0.2%-0.7%) | (0.5%-4.0%) | (0.5%-2.4%) |
|  | 2 | 0.7% | 0.8% | 1.9% |
|  |  | (0.4%-1.0%) | (0.0%-1.4%) | (0.7%-3.2%) |
|  | 3 | 0.7% | 1.0% | 0.8% |
|  |  | (0.4%-1.0%) | (0.0%-1.8%) | (0.1%-1.3%_ |
|  | 4 | 0.3% | 1.3% | 0.3% |
|  |  | (0.2%-0.5%) | (0.0%-2.4%) | (0.1%-0.8%) |
|  | 5 | 0.3% | 0.4% | 0.3% |
|  |  | (0.1%-0.5%) | (0.0%-0.7%) | (0.0%-0.5%) |
| India | 1 | 2.3% | 6.4% | 3.7% |
|  |  | (1.4%-3.2%) | (4.7%-7.9%) | (2.7%-4.8%) |
|  | 2 | 2.8% | 4.2% | 3.2% |
|  |  | (1.7%-4.0%) | (3.1%-5.1%) | (2.3%-4.1%) |
|  | 3 | 1.7% | 2.7% | 2.3% |
|  |  | (1.0%-2.5%) | (1.7%-3.7%) | (1.3%-3.2%) |
|  | 4 | 1.1% | 1.4% | 1.0% |
|  |  | (0.6%-1.6%) | (0.8%-2.1%) | (0.5%-1.6%) |
|  | 5 | 0.5% | 0.6% | 0.4% |
|  |  | (0.2%-1.0%) | (0.3%-1.0%) | (0.2%-0.7%) |
| Mexico | 1 | 3.5% | 0.8% | 0.5% |
|  |  | (0.5%-7.8%) | (0.1%-1.5%) | (0.0%-1.2%) |
|  | 2 | 0.8% | 0.9% | 0.8% |
|  |  | (0.2%-2.4%) | (0.0%-2.2%) | (0.2%-1.6%) |
|  | 3 | 3.8% | 3.9% | 0.6% |
|  |  | (0.4%-6.7%) | (0.0%-8.7%) | (0.1%-1.1%) |
|  | 4 | 1.0% | 0.4% | 1.1% |
|  |  | (0.3%-1.8%) | (0.0%-0.8%) | (0.0%-3.7%) |
|  | 5 | 1.5% | 0.0% | 0.1% |
|  |  | (0.2%-4.3%) | (0.0%-0.3%) | (0.0%-0.3%) |
| Russia | 1 | 0.8% | 0.0% | 0.1% |
|  |  | (0.4%-1.4%) | (0.0%-0.0%) | (0.0%-0.3%) |
|  | 2 | 3.0% | 0.0% | 0.7% |
|  |  | (1.3%-4.7%) | (0.0%-0.0%) | (0.0%-1.8%) |
|  | 3 | 1.4% | 0.3% | 0.0% |
|  |  | (0.4%-2.4%) | (0.0%-0.5%) | (0.0%-0.0%) |
|  | 4 | 0.1% | 0.4% | 0.0% |
|  |  | (0.0%-0.3%) | (0.0%-0.9%) | (0.0%-0.1%) |
|  | 5 | 0.1% | 0.7% | 0.1% |
|  |  | (0.0%-0.7%) | (0.0%-2.5%) | (0.0%-0.1%) |
| South Africa | 1 | 2.0% | 0.5% | 1.6% |
|  |  | (1.2%-2.9%) | (0.0%-1.2%) | (0.2%-2.8%) |
|  | 2 | 3.2% | 0.1% | 0.3% |
|  |  | (1.1%-6.0%) | (0.0%-0.2%) | (0.1%-0.5%) |
|  | 3 | 2.1% | 3.1% | 1.0% |
|  |  | (1.2%-3.7%) | (0.0%-6.7%) | (0.2%-3.0%) |
|  | 4 | 0.9% | 0.2% | 0.2% |
|  |  | (0.3%-1.7%) | (0.0%-0.4%) | (0.0%-0.5%) |
|  | 5 | 0.6% | 0.7% | 0.1% |
|  |  | (0.2%-1.4%) | (0.0%-2.2%) | (0.0%-0.4%) |

**Table S9: Catastrophic health expenditure (CHE) by disease area as a share of all CHE cases and wealth quintile by country.**

| Country | Wealth Quintile | NCDs | CDs | Other |
| --- | --- | --- | --- | --- |
| China | 1 | 29.1% | 28.2% | 42.7% |
|  |  | (22.7%-36.3%) | (21.7%-34.8%) | (34.4%-49.9%) |
|  | 2 | 39.5% | 16.4% | 44.1% |
|  |  | (28.9%-49.9%) | (11.1%-22.1%) | (34.4%-53.9%) |
|  | 3 | 36.1% | 22.3% | 41.6% |
|  |  | (27.5%-45.8%) | (10.9%-31.3%) | (31.7%-52.2%) |
|  | 4 | 34.4% | 16.8% | 48.8% |
|  |  | (25.9%-44.3%) | (8.8%-24.0%) | (36.4%-60.5%) |
|  | 5 | 59.1% | 6.4% | 34.5% |
|  |  | (42.8%-74.9%) | (2.8%-11.1%) | (18.3%-50.4%) |
| Ghana | 1 | 11.2% | 54.7% | 34.0% |
|  |  | (4.5%-25.6%) | (21.1%-78.6%) | (12.6%-62.8%) |
|  | 2 | 21.4% | 22.6% | 56.1% |
|  |  | (10.0%-48.5%) | (0.0%-50.5%) | (25.4%-83.3%) |
|  | 3 | 31.2% | 35.2% | 33.6% |
|  |  | (14.5%-69.0%) | (2.4%-67.9%) | (6.3%-64.1%) |
|  | 4 | 21.3% | 61.2% | 17.5% |
|  |  | (6.9%-65.6%) | (8.1%-86.1%) | (3.1%-62.1%) |
|  | 5 | 36.3% | 34.9% | 28.8% |
|  |  | (14.5%-84.5%) | (0.0%-68.3%) | (3.0%-67.1%) |
| India | 1 | 18.3% | 51.6% | 30.0% |
|  |  | (12.2%-25.8%) | (42.6%-60.4%) | (23.2%-37.7%) |
|  | 2 | 27.3% | 41.3% | 31.4% |
|  |  | (19.0%-37.1%) | (32.6%-48.9%) | (23.9%-39.3%) |
|  | 3 | 25.7% | 40.4% | 33.9% |
|  |  | (15.8%-37.2%) | (27.3%-53.0%) | (23.1%-44.9%) |
|  | 4 | 30.3% | 41.0% | 28.7% |
|  |  | (18.1%-44.1%) | (26.5%-56.0%) | (15.2%-41.9%) |
|  | 5 | 32.9% | 40.0% | 27.0% |
|  |  | (15.8%-54.1%) | (16.5%-60.1%) | (11.9%-45.5%) |
| Mexico | 1 | 66.0% | 21.7% | 12.3% |
|  |  | (26.4%-93.3%) | (2.0%-57.8%) | (0.3%-43.6%) |
|  | 2 | 32.3% | 32.8% | 34.9% |
|  |  | (10.2%-78.6%) | (0.0%-70.3%) | (8.1%-75.2%) |
|  | 3 | 49.7% | 40.0% | 10.3% |
|  |  | (5.8%-94.5%) | (0.3%-90.3%) | (1.1%-44.2%) |
|  | 4 | 47.7% | 15.6% | 36.6% |
|  |  | (12.2%-92.1%) | (0.0%-52.5%) | (3.3%-79.7%) |
|  | 5 | 86.9% | 1.6% | 11.5% |
|  |  | (48.6%-100.0%) | (0.0%-29.6%) | (0.0%-39.0%) |
| Russia | 1 | 84.8% | 1.8% | 13.4% |
|  |  | (65.4%-99.2%) | (0.0%-4.1%) | (0.0%-32.1%) |
|  | 2 | 80.1% | 0.0% | 19.9% |
|  |  | (55.7%-99.7%) | (0.0%-0.0%) | (0.3%-44.3%) |
|  | 3 | 83.1% | 15.7% | 1.1% |
|  |  | (48.9%-100.0%) | (0.0%-46.2%) | (0.0%-4.2%) |
|  | 4 | 41.7% | 52.1% | 6.3% |
|  |  | (6.0%-100.0%) | (0.0%-92.2%) | (0.0%-40.1%) |
|  | 5 | 29.7% | 55.2% | 15.1% |
|  |  | (3.1%-91.0%) | (0.0%-93.6%) | (0.0%-55.1%) |
| South Africa | 1 | 51.1% | 12.7% | 36.2% |
|  |  | (31.2%-86.7%) | (0.0%-31.6%) | (6.0%-58.9%) |
|  | 2 | 88.5% | 3.3% | 8.2% |
|  |  | (75.6%-96.1%) | (0.1%-8.7%) | (2.7%-19.3%) |
|  | 3 | 37.8% | 45.7% | 16.5% |
|  |  | (20.0%-84.9%) | (1.4%-71.3%) | (3.3%-51.8%) |
|  | 4 | 68.1% | 12.2% | 19.7% |
|  |  | (36.8%-89.9%) | (0.0%-34.2%) | (4.6%-41.5%) |
|  | 5 | 52.4% | 35.4% | 12.2% |
|  |  | (10.2%-92.2%) | (0.0%-84.7%) | (0.0%-39.4%) |

Appendix 4: Characterizing utilization intensity and spending patterns

Tables S10-S17 show the full regressions results for the coefficients shown in Figure 2 in the main text.

All regressions were conducted with the individual survey weights provided by SAGE. They were clustered at the household level and an offset of 10% of the mean (computed by country) was applied to all log-transformed variables to preserve zeros.

|  | **Table S10: Log Outpatient Spending (2017 Purchasing Power Parity)** | | | | | | |
| --- | --- | --- | --- | --- | --- | --- | --- |
|  | (1)  All | (2)  China | (3)  Ghana | (4)  India | (5)  Mexico | (6)  Russia | (7)  South Africa |
| CDs (Reference) |  |  |  |  |  |  |  |
| NCDs | 0.435*** | 0.422*** | -0.0841 | 0.428*** | -0.477 | 0.121 | 0.116 |
| Injury | 0.515*** | 0.706** | 0.519* | 0.502** | -2.311*** | -0.812* | -0.0906 |
| Pain | 0.369*** | 0.423*** | -0.0801 | 0.368*** | -0.458 | -0.444 | 1.548*** |
| Surgery | 0.899*** | 0.817*** | 0.508 | 1.131*** | -0.497 | -0.306 | 0.0746 |
| Other | 0.698*** | 1.036*** | -0.0109 | 0.500*** | -0.346 | -0.134 | 0.888* |
| Unidentified | 0.508*** | 0.603*** | -0.0295 | 0.379*** | -0.670* | 0.0328 | 0.302 |
| China (Reference) |  |  |  |  |  |  |  |
| India | -0.368*** | |  |  |  |  |  |
| Mexico | -0.0515 |  |  |  |  |  |  |
| Russia | -0.357* |  |  |  |  |  |  |
| South Africa | -0.465** |  |  |  |  |  |  |
| Ghana | -0.782*** | |  |  |  |  |  |
| Urban | 0.127* | 0.337** | 0.292* | -0.0483 | 0.223 | 0.411 | -0.214 |
| No Schooling (Reference) |  |  |  |  |  |  |  |
| Primary School | -0.0111 | 0.0596 | -0.102 | -0.0562 | 0.0482 | 0.387 | 0.162 |
| Secondary School | -0.00890 | -0.0266 | 0.230 | 0.0640 | 0.227 | -0.0551 | 1.238*** |
| College | 0.0230 | -0.0539 | -0.125 | 0.0329 | 0.478 | 0.0616 | 0.747* |
| Post-College | -0.555** |  | -0.630 |  |  | -1.251** |  |
| WQ 1 (Reference) |  |  |  |  |  |  |  |
| WQ 2 | 0.190*** | 0.124 | 0.158 | 0.202*** | -0.653** | 0.533 | 0.586* |
| WQ 3 | 0.190*** | 0.134 | 0.155 | 0.184** | 1.052** | 0.909*** | -0.0809 |
| WQ 4 | 0.172** | 0.0437 | 0.169 | 0.283*** | 0.180 | 0.259 | -0.0335 |
| WQ 5 | 0.304*** | 0.114 | 0.192 | 0.428*** | -0.191 | 0.740* | -0.0194 |
| Female | 0.0166 | 0.0310 | 0.0397 | 0.0321 | 0.0244 | -0.131 | -0.615* |
| Age | 0.000553 | 0.000694 | -0.00539 | 0.000209 | 0.000568 | -0.00365 | 0.0191** |
| Constant | 2.907*** | 2.909*** | 2.643*** | 2.567*** | 3.433*** | 2.606*** | 1.948*** |
| N | 19497 | 6367 | 2691 | 8005 | 532 | 1289 | 613 |
|  | * p<0.05, ** p<0.01, *** p<0.001 | | | | | | |

|  | **Table S11: Log Inpatient Spending (2017 PPP)** | | | | | | |
| --- | --- | --- | --- | --- | --- | --- | --- |
|  | (1)  All | (2)  China | (3)  Ghana | (4)  India | (5)  Mexico | (6)  Russia | (7)  South Africa |
| CDs (Reference) |  |  |  |  |  |  |  |
| NCDs | 0.481*** | 0.448* | -0.136 | 0.563** | -1.351 | 0.779* | -1.539** |
| Injury | 0.639** | 0.718* | 0.428 | 0.458 | -2.418* | 0.723 | 1.390** |
| Pain | 0.0260 | 0.0113 | 0.504 | 0.260 | -0.0731 | -0.289 | -1.268* |
| Surgery | 0.488*** | 0.401 | 0.209 | 0.586** | -0.481 | 1.233** | -1.797*** |
| Other | -0.322 | -0.506 | 0.784 | 0.260 | -1.495* | 0.209 | -1.820*** |
| Unidentified | 0.349*** | 0.388 | -0.160 | 0.311* | -1.306 | 0.368 | -1.173*** |
| China (Reference) |  |  |  |  |  |  |  |
| India | -0.933*** |  |  |  |  |  |  |
| Mexico | -0.732* |  |  |  |  |  |  |
| Russia | -2.769*** |  |  |  |  |  |  |
| South Africa | -0.344 |  |  |  |  |  |  |
| Ghana | -2.745*** |  |  |  |  |  |  |
| Urban | 0.225* | 0.279* | 0.760*** | 0.173 | -1.340*** | -0.305 | -0.370 |
| No Schooling (Reference) |  |  |  |  |  |  |  |
| Primary School | -0.0911 | -0.160 | -0.223 | -0.0560 | 0.986 | 0.463 | 0.201 |
| Secondary School | -0.123 | -0.160 | 0.696 | -0.0662 | -0.991 | -0.327 | -0.0563 |
| College | -0.126 | -0.164 | -0.212 | -0.211 | 0.462 | 0.209 | 0.264 |
| Post-College | 0.850 |  | 1.469** |  |  |  |  |
| WQ 1 (Reference) |  |  |  |  |  |  |  |
| WQ 2 | 0.149 | 0.104 | 0.227 | 0.173 | 0.507 | 0.108 | 0.715 |
| WQ 3 | 0.103 | -0.130 | -0.205 | 0.420** | 0.398 | 0.00627 | 0.946*** |
| WQ 4 | 0.257* | 0.0779 | 0.190 | 0.635*** | 0.589 | 0.0181 | 1.485*** |
| WQ 5 | 0.438*** | 0.232 | 0.267 | 0.743*** | -0.162 | 0.473 | 1.878** |
| Female | -0.166 | -0.200 | 0.132 | -0.235 | -0.816* | 0.345 | -0.0675 |
| Age | -0.0058* | -0.00404 | -0.00868 | -0.011*** | -0.00597 | -0.00457 | 0.00950 |
| Constant | 6.858*** | 6.946*** | 3.936*** | 5.963*** | 8.307*** | 3.783*** | 5.825*** |
| N | 3464 | 1538 | 327 | 986 | 68 | 425 | 120 |
| * p<0.05, ** p<0.01, *** p<0.001 | | | | | | | |

|  | **Table S12: Log Outpatient Drug Spending (2017 PPP)** | | | | | | |
| --- | --- | --- | --- | --- | --- | --- | --- |
|  | (1)  All | (2)  China | (3)  Ghana | (4)  India | (5)  Mexico | (6)  Russia | (7)  South Africa |
| CDs (Reference) |  |  |  |  |  |  |  |
| NCDs | 0.251*** | 0.234* | -0.217 | 0.303*** | -0.270 | -0.796** | 0.614* |
| Injury | 0.469** | 0.549** | 0.343 | 0.493* | -0.766 | -1.113** | 0.0699 |
| Pain | 0.246*** | 0.176 | -0.0453 | 0.330*** | 0.330 | -1.087* | -0.318 |
| Surgery | 0.725*** | 0.564* | -0.115 | 1.034*** | -0.197 | -1.640*** | -0.290 |
| Other | 0.505*** | 0.616* | -0.196 | 0.426*** | -0.803** | -0.270 | -0.377 |
| Unidentified | 0.287*** | 0.294 | -0.516* | 0.266*** | 0.110 | -0.385 | -0.0248 |
| China (Reference) |  |  |  |  |  |  |  |
| India | -0.511*** |  |  |  |  |  |  |
| Mexico | 0.469* |  |  |  |  |  |  |
| Russia | -0.880*** |  |  |  |  |  |  |
| South Africa | -2.909*** |  |  |  |  |  |  |
| Ghana | -1.130*** |  |  |  |  |  |  |
| Urban | 0.0855 | 0.331* | 0.254 | -0.0786 | 0.0899 | 0.172 | -0.443 |
| No Schooling (Reference) |  |  |  |  |  |  |  |
| Primary School | -0.0424 | -0.0143 | 0.0684 | -0.0861 | -0.297 | 0.673 | 0.0289 |
| Secondary School | -0.0869 | -0.238** | 0.226 | 0.0549 | 0.00963 | 0.279 | 1.117* |
| College | -0.0252 | -0.179 | -0.0803 | -0.0111 | 0.0973 | -0.116 | 0.604 |
| Post-College | -1.185*** |  | -1.634*** |  |  | -2.152** |  |
| WQ 1 (Reference) |  |  |  |  |  |  |  |
| WQ 2 | 0.159*** | 0.152 | 0.133 | 0.160* | -0.212 | 0.295 | -0.707 |
| WQ 3 | 0.133* | 0.0869 | 0.133 | 0.145* | 0.682** | 0.638** | -0.418 |
| WQ 4 | 0.163* | 0.169 | -0.0231 | 0.197** | -0.172 | -0.0743 | -0.246 |
| WQ 5 | 0.226*** | 0.212 | 0.106 | 0.280*** | 0.0776 | 0.0862 | -0.113 |
| Female | 0.0281 | -0.0383 | 0.0163 | 0.0530 | -0.432 | 0.222 | 0.0425 |
| Age | 0.000563 | 0.000521 | -0.00365 | -0.000571 | 0.000945 | 0.000883 | 0.00489 |
| Constant | 2.726*** | 2.784*** | 2.007*** | 2.247*** | 3.632*** | 2.574*** | -0.0563 |
| N | 18095 | 5584 | 2471 | 7968 | 247 | 1234 | 591 |
| * p<0.05, ** p<0.01, *** p<0.001 | | | | | | | |

|  | **Table S13: Log Inpatient Drug Spending (2017 PPP)** | | | | | | |
| --- | --- | --- | --- | --- | --- | --- | --- |
|  | (1)  All | (2)  China | (3)  Ghana | (4)  India | (5)  Mexico | (6)  Russia | (7)  South Africa |
| CDs (Reference) |  |  |  |  |  |  |  |
| NCDs | 0.467** | 0.518* | -0.650 | 0.518** | 1.068 | 0.527 | 0.0359 |
| Injury | 0.585* | 0.700 | 0.690 | 0.508* |  | 1.387* | -0.0211 |
| Pain | -0.0926 | 0.0832 | 0.281 | 0.282 | 1.135 | -0.647 | 0.0111 |
| Surgery | 0.292 | 0.398* | 0.389 | 0.418 | 1.698 | -0.359 | -0.0303 |
| Other | -0.119 | -0.319 | 1.844* | 0.222 | 3.502** | -0.340 | 0.0174 |
| Unidentified | 0.245* | 0.340 | -0.536 | 0.229 | 0.584 | -0.0125 | -0.0229 |
| China (Reference) |  |  |  |  |  |  |  |
| India | -1.059*** | |  |  |  |  |  |
| Mexico | -0.763*** | |  |  |  |  |  |
| Russia | -3.551*** | |  |  |  |  |  |
| South Africa | -1.089*** | |  |  |  |  |  |
| Ghana | -3.916*** | |  |  |  |  |  |
| Urban | 0.254* | 0.229 | 0.791* | 0.317* | 0.545 | -0.431 | 0.0306 |
| No Schooling (Reference) |  |  |  |  |  |  |  |
| Primary School | -0.304* | -0.555** | -0.162 | -0.0893 | 0.639 | -0.403 | 0.0935 |
| Secondary School | -0.160 | -0.164 | -0.640 | -0.267 | 0.135 | 0.0944 | -0.0129 |
| College | -0.158 | -0.151 | -0.0927 | -0.162 | 2.153 | 0.180 | 0.00249 |
| Post-College | 0.657* |  | 1.822*** |  |  |  |  |
| WQ 1 (Reference) |  |  |  |  |  |  |  |
| WQ 2 | 0.224 | 0.343 | 0.151 | 0.165 | 2.026* | -0.287 | 0.0642 |
| WQ 3 | 0.0866 | -0.205 | 0.0218 | 0.367 | 0.310 | -0.235 | 0.218** |
| WQ 4 | 0.226 | 0.107 | -0.0228 | 0.515** | 0.483 | -0.402 | 0.0740 |
| WQ 5 | 0.367** | 0.176 | -0.620 | 0.545*** | -0.190 | 0.226 | 0.0535 |
| Female | -0.238* | -0.269 | -0.706* | -0.221 | 0.344 | -0.0547 | -0.00881 |
| Age | -0.00624 | -0.00341 | -0.0119 | -0.0126*** | -0.0149 | 0.00330 | -0.000891 |
| Constant | 6.409*** | 6.379*** | 3.221*** | 5.382*** | 3.711 | 3.032*** | 5.162*** |
| N | 2880 | 1070 | 284 | 962 | 26 | 421 | 117 |
|  | * p<0.05, ** p<0.01, *** p<0.001 | | | | | | |

|  | **Table S14: Outpatient Drug Spending / Total Visit Spend** | | | | | | |
| --- | --- | --- | --- | --- | --- | --- | --- |
|  | (1)  All | (2)  China | (3)  Ghana | (4)  India | (5)  Mexico | (6)  Russia | (7)  South Africa |
| CDs (Reference) |  |  |  |  |  |  |  |
| NCDs | -0.096*** | -0.093*** | -0.085 | -0.088*** | -0.136* | -0.490*** | 0.145* |
| Injury | -0.067 | -0.178** | -0.084 | -0.0165 | 0.041 | -0.250** | 0.029 |
| Pain | -0.067*** | -0.121** | 0.082 | -0.0237 | 0.153 | -0.709*** | -0.090 |
| Surgery | -0.056* | -0.059 | -0.044 | -0.066 | -0.354** | -0.752** | -0.126 |
| Other | -0.118** | -0.211** | -0.120 | -0.073* | 0.037 | -0.178 | -0.107 |
| Unidentified | -0.115*** | -0.161*** | -0.249*** | -0.059** | -0.078 | -0.460*** | 0.030 |
| China (Reference) |  |  |  |  |  |  |  |
| India | -0.196*** |  |  |  |  |  |  |
| Mexico | -0.230*** |  |  |  |  |  |  |
| Russia | -0.230** |  |  |  |  |  |  |
| South Africa | -0.729*** |  |  |  |  |  |  |
| Ghana | -0.259*** |  |  |  |  |  |  |
| Urban | -0.066*** | -0.108*** | -0.004 | -0.0439* | -0.029 | 0.003 | -0.094 |
| No Schooling (Reference) |  |  |  |  |  |  |  |
| Primary School | -0.002 | -0.008 | 0.036 | -0.011 | 0.139 | 0.220 | -0.020 |
| Secondary School | -0.013 | -0.048** | -0.053 | 0.004 | -0.116 | 0.298* | 0.125 |
| College | -0.030 | -0.031 | -0.020 | -0.018 | 0.196* | -0.093 | 0.068 |
| Post-College | -0.537*** |  | -0.559*** |  |  |  |  |
| WQ 1 (Reference) |  |  |  |  |  |  |  |
| WQ 2 | -0.039* | -0.016 | -0.003 | -0.039 | -0.086 | -0.238 | -0.0435 |
| WQ 3 | -0.040* | -0.013 | 0.0004 | -0.043* | -0.181** | -0.314 | 0.0735 |
| WQ 4 | -0.0486** | 0.033 | -0.112 | -0.0939*** | -0.0167 | -0.367* | 0.0588 |
| WQ 5 | -0.070*** | 0.010 | -0.072 | -0.102*** | -0.190* | -0.428* | 0.104 |
| Female | -0.003 | -0.032* | -0.050 | 0.010 | -0.057 | 0.132 | 0.102 |
| Age | 0.0001 | 0.0007 | -0.0007 | -0.0003 | 0.001 | 0.002 | -0.002 |
| Constant | 1.002*** | 0.973*** | 0.750*** | 0.805*** | 0.673*** | 1.223*** | 0.0135 |
| N | 17017 | 5387 | 2205 | 7905 | 245 | 859 | 416 |
|  | * p<0.05, ** p<0.01, *** p<0.001 | | | | | | |

|  | **Table S15: Inpatient Drug Spending / Total Visit Spending** | | | | | | | |
| --- | --- | --- | --- | --- | --- | --- | --- | --- |
|  | | (1)  All | (2)  China | (3)  Ghana | (4)  India | (5)  Mexico | (6)  Russia | (7)  South Africa |
| CDs (Reference) | |  |  |  |  |  |  |  |
| NCDs | | -0.092* | -0.165 | -0.154 | -0.050 | -0.402 | -0.266 | 0.010 |
| Injury | | -0.046 | -0.133 | 0.252 | 0.004 |  | 0.274 | 0.020 |
| Pain | | -0.165* | -0.158 | 0.074 | -0.00200 | 0.0823 | -0.511* | -0.007 |
| Surgery | | -0.125** | -0.114 | 0.047 | -0.122 | -0.385 | -0.599*** | 0.010 |
| Other | | 0.003 | 0.021 | 0.355* | -0.110 | 0.090 | -0.392* | 0.101 |
| Unidentified | | -0.126** | -0.208 | -0.108 | -0.067 | -0.181 | -0.239 | 0.006 |
| China (Reference) | |  |  |  |  |  |  |  |
| India | | -0.067 |  |  |  |  |  |  |
| Mexico | | -0.104 |  |  |  |  |  |  |
| Russia | | -0.141 |  |  |  |  |  |  |
| South Africa | | -0.629*** |  |  |  |  |  |  |
| Ghana | | -0.313*** |  |  |  |  |  |  |
| Urban | | 0.028 | -0.026 | 0.166* | 0.090* | 0.541** | -0.055 | -0.003 |
| No Schooling (Reference) | |  |  |  |  |  |  |  |
| Primary School | | -0.092* | -0.153** | -0.036 | -0.020 | -0.176 | -0.333* | 0.006 |
| Secondary School | | -0.024 | -0.031 | -0.128 | -0.063 | -0.508 | 0.039 | 0.012 |
| College | | -0.021 | 0.038 | -0.021 | -0.010 | -0.434 | -0.173 | -0.001 |
| Post-College | | 0.002 |  | 0.116 |  |  |  |  |
| WQ 1 (Reference) | |  |  |  |  |  |  |  |
| WQ 2 | | 0.003 | 0.0455 | 0.040 | -0.008 | -0.043 | -0.0751 | -0.004 |
| WQ 3 | | -0.022 | -0.007 | 0.055 | -0.032 | -0.294 | -0.181 | 0.094*** |
| WQ 4 | | -0.029 | 0.0121 | -0.035 | -0.050 | -0.152 | -0.227 | 0.010 |
| WQ 5 | | -0.052 | -0.026 | -0.135 | -0.0945 | -0.347 | -0.224* | 0.010 |
| Female | | -0.014 | -0.004 | -0.155* | 0.017 | 0.094 | -0.107 | 0.008 |
| Age | | 0.001 | 0.001 | -0.002 | 0.0001 | -0.004 | 0.005 | -0.001 |
| Constant | | 0.674*** | 0.709*** | 0.411* | 0.584*** | 0.767 | 0.944** | 0.037 |
| N | | 2610 | 1046 | 261 | 952 | 25 | 265 | 61 |
|  | * p<0.05, ** p<0.01, *** p<0.001 | | | | | | | |

|  | **Table S16: Outpatient: probability of utilizing a private facility** | | | | | | | |
| --- | --- | --- | --- | --- | --- | --- | --- | --- |
|  | | (1)  All | (2)  China | (3)  Ghana | (4)  India | (5)  Mexico | (6)  Russia | (7)  South Africa |
| CDs  (Reference) | |  |  |  |  |  |  |  |
| NCDs | | -0.008 | -0.095* | -0.063 | 0.056* | -0.132 | 0.141 | 0.026 |
| Injury | | -0.046 | -0.287** | 0.017 | 0.045 | -0.428* | -0.0267 | 0.094 |
| Pain | | 0.011 | 0.003 | -0.057 | 0.005 | 0.057 | -0.012 | 0.369* |
| Surgery | | -0.044 | -0.185** | -0.004 | 0.202* | -0.047 | 0.251 | -0.321* |
| Other | | -0.047 | -0.151* | 0.479*** | 0.071 | 0.135 | -0.026 | 0.204 |
| Unidentified | | -0.094*** | -0.151*** | -0.026 | -0.029 | -0.101 | 0.0008 | 0.207 |
| China (Reference) | |  |  |  |  |  |  |  |
| India | | 0.334*** |  |  |  |  |  |  |
| Mexico | | 0.066 |  |  |  |  |  |  |
| Russia | | -0.176*** |  |  |  |  |  |  |
| South Africa | | 0.0169 |  |  |  |  |  |  |
| Ghana | | -0.116** |  |  |  |  |  |  |
| Urban | | -0.056 | -0.138** | 0.036 | 0.055 | 0.111 | 0.075 | -0.0002 |
| No Schooling (Reference) | |  |  |  |  |  |  |  |
| Primary School | | -0.006 | -0.057 | -0.043 | -0.005 | -0.197* | -0.018 | 0.110 |
| Secondary School | | -0.003 | -0.068 | -0.028 | 0.041 | -0.128 | -0.032 | 0.141 |
| College | | 0.0007 | -0.113* | -0.026 | 0.079* | -0.193 | 0.034 | 0.324** |
| Post-College | | -0.0972 |  | -0.096* |  |  | -0.224* |  |
| Graduate | | -0.125** |  |  |  |  | 0.025 |  |
| WQ 1 (Reference) | |  |  |  |  |  |  |  |
| WQ 2 | | 0.080** | -0.018 | -0.061 | 0.102** | -0.0482 | 0.093 | 0.058 |
| WQ 3 | | 0.068* | -0.090 | 0.035 | 0.108** | 0.139 | 0.0052 | -0.029 |
| WQ 4 | | 0.042 | -0.185** | 0.075 | 0.146*** | 0.230 | 0.044 | 0.038 |
| WQ 5 | | 0.012 | -0.263*** | 0.094 | 0.167*** | 0.110 | 0.120 | 0.203 |
| Female | | 0.008 | -0.006 | -0.003 | 0.057* | -0.130 | -0.089 | -0.133 |
| Age | | -0.002** | -0.004*** | -0.0005 | -0.001* | 0.0004 | -0.002 | 0.003 |
| Constant | | 0.363*** | 0.797*** | 0.183** | 0.509*** | 0.410 | 0.00454 | -0.0354 |
| N | | 23150 | 6640 | 2962 | 8436 | 991 | 2446 | 1675 |
|  | * p<0.05, ** p<0.01, *** p<0.001 | | | | | | | |
|  |  | | | | | | | |
|  | **Table S17: Inpatient: probability of utilizing a private facility** | | | | | | | |
|  | | (1)  All | (2)  China | (3)  Ghana | (4)  India | (5)  Mexico | (6)  Russia | (7)  South Africa |
| CDs (Reference) | |  |  |  |  |  |  |  |
| NCDs | | 0.0003 | -0.022 | 0.198 | 0.019 | -0.002 | 0.041 | -0.524*** |
| Injury | | 0.032 | 0.070 | -0.177 | -0.114 | -0.307 | 0.011 | -0.263* |
| Pain | | 0.025 | -0.013 | -0.043 | 0.071 | -0.0491 | -0.007 | -0.297** |
| Surgery | | 0.015 | -0.014 | -0.196 | 0.031 | -0.342* | 0.057 | -0.009 |
| Other | | -0.058 | -0.060 | -0.197 | -0.137 | -0.070 | 0.021 | 0.723* |
| Unidentified | | -0.041 | -0.047 | 0.033 | -0.056 | 0.032 | 0.021 | -0.250** |
| China (Reference) | |  |  |  |  |  |  |  |
| India | | 0.570*** |  |  |  |  |  |  |
| Mexico | | 0.156 |  |  |  |  |  |  |
| Russia | | -0.045* |  |  |  |  |  |  |
| South Africa | | 0.243** |  |  |  |  |  |  |
| Ghana | | 0.231*** |  |  |  |  |  |  |
| Urban | | 0.022 | -0.005 | -0.0147 | 0.067 | 0.207 | 0.0005 | 0.156* |
| No Schooling (Reference) | |  |  |  |  |  |  |  |
| Primary School | | -0.049 | -0.022 | -0.261* | -0.103 | 0.065 | -0.003 | -0.101 |
| Secondary School | | -0.009 | 0.005 | -0.0318 | -0.013 | -0.446 | -0.005 | -0.198 |
| College | | -0.038 | -0.005 | -0.0203 | -0.109 | -0.217 | 0.004 | 0.302** |
| Post-College | | -0.356*** |  | -0.417** |  |  |  |  |
|  | |  |  |  |  |  |  |  |
| WQ 1 (Reference) | |  |  |  |  |  |  |  |
| WQ 2 | | 0.015 | 0.014 | 0.0978 | -0.027 | 0.008 | 0.05 | 0.086 |
| WQ 3 | | 0.049 | -0.005 | 0.0420 | 0.110 | 0.031 | 0.004 | 0.432** |
| WQ 4 | | 0.022 | -0.042 | 0.190 | 0.10 | 0.090 | 0.002 | 0.430** |
| WQ 5 | | 0.097* | -0.004 | 0.312* | 0.270*** | -0.122 | 0.009 | 0.473*** |
| Female | | 0.006 | -0.005 | -0.0986 | 0.006 | -0.013 | 0.017 | 0.187* |
| Age | | -0.001 | -0.0008 | -0.00302 | -0.002 | 0.002 | -0.001 | 0.004 |
| Constant | | 0.086 | 0.122 | 0.405* | 0.627*** | 0.194 | 0.029 | -0.400 |
| N | | 3989 | 1571 | 366 | 1035 | 99 | 706 | 212 |
|  | * p<0.05, ** p<0.01, *** p<0.001 | | | | | | | |

**Table S18: Estimated number of visits to catastrophic health expenditure (CHE) by disease area in China, Ghana, India, Mexico, Russia, and South Africa. Uncertainty intervals in parentheses.**

| **Number of visits to CHE** | **NCD**  **CHE Cases** | **CD CHE cases** | **Injury**  **CHE cases** |
| --- | --- | --- | --- |
| 1 | 25%  (23% - 27%) | 29%  (27% - 31%) | 51%  (44% - 59%) |
| 2 | 6%  (5% - 7%) | 8%  (7% - 9%) | 4%  (2% - 7%) |
| 3 | 5%  (4% - 5%) | 6%  (5% - 7%) | 4%  (<1% - 7%) |
| 4 | 4%  (3% - 5%) | 5%  (3% -6%) | 2%  (<1% - 5%) |
| 5 | 61%  (58% - 64%) | 53%  (50% - 55%) | 39%  (32% - 46%) |

Note: The number of visits to CHE captures the cumulative number of visits that occurred before CHE took place. Visits were ranked by the most expensive to the least expensive. Out-of-pocket spending was then calculated cumulatively, based on the rank-order of visits, and then compared with capacity-to-pay to assess how many visits occurred before spending was pushed across the 40% capacity to pay CHE threshold. The objective was to assess whether there were differences by disease area in the share of CHE cases that arose because of one, large spending visit (shock) versus the cumulation of spending over many visits. Differences for non-communicable disease (NCD) and communicable disease (CD) care were robust to controlling for country, age, sex, education, quintile and residence, and the total number of inpatient and outpatient visits in the last 12 months using ordinary least squares regression. All results presented with survey weights to depict results to be nationally representative.

Tables S19-S23 show the regression results for CHE cases that only examined: 1) the outpatient spending share out of total health spending, 2) the number of outpatient visits, and 3) the number of visits that occurred in order to push OOP health spending over the 40% capacity-to-pay threshold, which we called the number of “visits-to-CHE”.

|  | **Table S19: Outpatient expenditure as a share of total health expenditure** | | | | | | |
| --- | --- | --- | --- | --- | --- | --- | --- |
|  | (1)  All | (2)  China | (3)  Ghana | (4)  India | (5) Mexico | (6)  Russia | (7)  South Africa |
| CD CHE case (Reference) |  |  |  |  |  |  |  |
| NCD CHE case | -0.0444 | -0.101 | -0.0191 | -0.00480 | -0.227* | 0.190 | 0.309* |
| Injury CHE case | -0.364*** | -0.523*** | -0.0840 | -0.00649 | 0 | -0.308* | 0.230 |
| Other CHE case | -0.155*** | -0.231*** | -0.0719 | -0.0799 | -0.173 | -0.381* | 0.0266 |
| China (Reference) |  |  |  |  |  |  |  |
| Ghana | 0.346*** |  |  |  |  |  |  |
| India | 0.225*** |  |  |  |  |  |  |
| Mexico | 0.288** |  |  |  |  |  |  |
| Russia | 0.288** |  |  |  |  |  |  |
| South Africa | 0.335*** |  |  |  |  |  |  |
| Age | 0.00234 | 0.00388 | 0.00425* | 0.000729 | -0.000360 | 0.00205 | -0.00444* |
| Urban residence | -0.128* | -0.0638 | 0.0634 | -0.194 | 0.0141 | -0.250* | -0.0762 |
| Female | 0.0915* | 0.134* | -0.0695 | 0.0572 | 0.0791 | -0.135 | -0.273 |
| No Schooling (Reference) |  |  |  |  |  |  |  |
| Primary School | 0.0494 | 0.0734 | 0.340** | 0.0354 | -0.202 | 0.440 | -0.0762 |
| Secondary School | -0.0565 | -0.0103 | 0.229 | -0.120 | -0.237* | 0.629*** | -0.0919 |
| College | -0.0248 | -0.0811 | 0.265** | 0.0623 | -0.770*** | 0.605** | -0.242 |
| Post-College | 0.0261 |  | 0.201** |  |  |  |  |
| WQ1 |  |  |  |  |  |  |  |
| WQ 2 | -0.0160 | 0.0198 | 0.0319 | -0.0441 | 0.216 | -0.0249 | 0.0304 |
| WQ 3 | 0.0158 | 0.0847 | 0.00528 | -0.0533 | 0.520*** | -0.215 | -0.0598 |
| WQ 4 | -0.0306 | 0.0544 | 0.0237 | -0.198** | 0.274 | 0.238 | -0.255* |
| WQ 5 | -0.114 | -0.0822 | 0.0543 | -0.184 | 0.531*** | 0.351 | -0.295*** |
| Constant | 0.519*** | 0.406* | 0.504* | 0.826*** | 0.778** | 0.247 | 1.266*** |
| N | 3226 | 1548 | 187 | 895 | 183 | 95 | 318 |

|  | **Table S20: Log number of outpatient visits** | | | | | | |
| --- | --- | --- | --- | --- | --- | --- | --- |
|  | (1)  All | (2)  China | (3)  Ghana | (4)  India | (5) Mexico | (6)  Russia | (7)  South Africa |
| CD CHE case (Reference) |  |  |  |  |  |  |  |
| NCD CHE case | 0.576** | 0.590* | 0.998** | 0.401* | 0.253 | 1.154 | 1.792** |
| Injury CHE case | -1.413*** | -1.818*** | 1.023* | -0.138 | 0 | 0.570 | 0.431 |
| Other CHE case | -0.128 | -0.414* | 0.887* | 0.290* | 0.394 | -0.621 | 0.484 |
| China (Reference) |  |  |  |  |  |  |  |
| Ghana | 1.035*** |  |  |  |  |  |  |
| India | 1.283*** |  |  |  |  |  |  |
| Mexico | 1.719*** |  |  |  |  |  |  |
| Russia | 0.745 |  |  |  |  |  |  |
| South Africa | 1.056** |  |  |  |  |  |  |
| Age | 0.0168** | 0.0309*** | 0.00182 | 0.00178 | -0.00898 | 0.0277 | -0.00257 |
| Urban residence | -0.524* | -0.548 | -0.0880 | -0.315 | -0.409 | -1.279 | -0.239 |
| Female | 0.297 | 0.630** | -0.00701 | -0.0839 | 0.452 | -0.509 | -0.811 |
| No Schooling (Reference) |  |  |  |  |  |  |  |
| Primary School | 0.227 | 0.489 | 1.054** | -0.0791 | -0.763 | 1.794 | 0.0424 |
| Secondary School | -0.0640 | 0.187 | -0.389 | 0.135 | 0.484 | 2.237** | -0.490 |
| College | 0.0576 | 0.102 | 1.343*** | 0.195 | -3.006*** | 3.296** | -1.128 |
| Post-College | 0.0893 |  | 1.312*** |  |  |  |  |
| WQ1 |  |  |  |  |  |  |  |
| WQ 2 | -0.0328 | 0.135 | 0.322 | -0.0564 | -0.250 | -0.203 | 0.0610 |
| WQ 3 | 0.578** | 1.125*** | 0.606* | -0.0856 | 0.356 | -0.358 | -0.631 |
| WQ 4 | 0.333 | 0.804 | 0.452 | -0.444 | -0.0897 | 0.436 | -0.441 |
| WQ 5 | 0.117 | 0.431 | 1.422*** | -0.396 | 0.113 | 2.393* | -1.165** |
| Constant | -0.896 | -2.114* | -0.553 | 1.276*** | 2.081* | -3.105 | 1.764* |
| N | 3226 | 1548 | 187 | 895 | 183 | 95 | 318 |
|  | * p<0.05, ** p<0.01, *** p<0.001 | | | | | | |

Note: Offset with 10% of the pooled mean to retain zero values.

|  | **Table S21: Log number of inpatient visits** | | | | | | |
| --- | --- | --- | --- | --- | --- | --- | --- |
|  | (1)  All | (2)  China | (3)  Ghana | (4)  India | (5) Mexico | (6)  Russia | (7)  South Africa |
| CD CHE case (Reference) |  |  |  |  |  |  |  |
| NCD CHE case | 0.141* | 0.221** | 0.0301 | 0.0360 | 0.481** | 0.332 | -0.588 |
| Injury CHE case | 0.276*** | 0.399*** | 0.379* | -0.0114 | 0 | 0.586* | -0.947* |
| Other CHE case | 0.127** | 0.176** | 0.178 | 0.0839 | 0.302 | 0.838** | -0.0763 |
| China (Reference) |  |  |  |  |  |  |  |
| Ghana | -0.235** |  |  |  |  |  |  |
| India | -0.158** |  |  |  |  |  |  |
| Mexico | -0.144 |  |  |  |  |  |  |
| Russia | -0.0951 |  |  |  |  |  |  |
| South Africa | -0.0977 |  |  |  |  |  |  |
| Age | -0.00146 | -0.00343 | -0.00404 | 0.000801 | -0.00425 | -0.0206* | 0.00750 |
| Urban residence | 0.0795 | 0.00979 | -0.135 | 0.157 | 0.177 | 0.275 | 0.228 |
| Female | -0.0550 | -0.0905 | 0.177* | -0.0230 | -0.151 | -0.0324 | 0.736 |
| No Schooling (Reference) |  |  |  |  |  |  |  |
| Primary School | -0.0585 | -0.0874 | -0.220 | -0.0346 | 0.233 | -1.543** | 0.0792 |
| Secondary School | 0.0747 | 0.0285 | -0.605* | 0.152 | 0.515** | -1.601** | 0.351* |
| College | 0.135 | 0.198 | -0.293** | 0.0414 | 0.772* | -1.890*** | 0.730** |
| Post-College | -0.250** |  | -0.291** |  |  |  |  |
| WQ1 |  |  |  |  |  |  |  |
| WQ 2 | -0.00216 | -0.0206 | -0.0195 | 0.00906 | -0.543** | 0.126 | 0.0709 |
| WQ 3 | 0.0561 | 0.0259 | 0.147 | 0.0650 | -1.035*** | 0.844** | 0.143 |
| WQ 4 | 0.0308 | -0.0352 | 0.00316 | 0.189* | -0.545* | -0.170 | 0.311* |
| WQ 5 | 0.101 | 0.0768 | 0.328* | 0.182 | -1.139*** | -0.194 | 0.262 |
| Constant | 0.197 | 0.322 | 0.196 | -0.0596 | 0.402 | 2.582*** | -0.762* |
| N | 3226 | 1548 | 187 | 895 | 183 | 95 | 318 |
|  | * p<0.05, ** p<0.01, *** p<0.001 | | | | | | |

Note: Offset with 10% of the pooled mean to retain zero values.

|  | **Table S22: Probability of one visit to catastrophic health expenditure (CHE)** | | | | | | | |
| --- | --- | --- | --- | --- | --- | --- | --- | --- |
|  | (1)  All | (2)  China | (3)  Ghana | (4)  India | (5) Mexico | (6)  Russia | (7)  South Africa |
| CD CHE case (Reference) |  |  |  |  |  |  |  |
| NCD CHE case | -0.109* | -0.0507 | -0.256* | -0.144* | 0.507** | -0.263 | -0.0833 |
| Injury CHE case | 0.244*** | 0.361*** | -0.0262 | 0.0515 | 0 | -0.610* | 0.577** |
| Other CHE case | -0.0265 | 0.0675 | -0.499*** | -0.108 | 0.243 | -0.211 | -0.0409 |
| China (Reference) |  |  |  |  |  |  |  |
| Ghana | -0.321*** |  |  |  |  |  |  |
| India | -0.233*** |  |  |  |  |  |  |
| Mexico | -0.106 |  |  |  |  |  |  |
| Russia | -0.165 |  |  |  |  |  |  |
| South Africa | -0.338*** |  |  |  |  |  |  |
| Age | -0.00188 | -0.002 | 0.004 | -0.001 | 0.0007 | 0.0002 | 0.0001 |
| Urban residence | 0.127* | 0.0355 | 0.230 | 0.231* | 0.379** | 0.166 | -0.0889 |
| Female | -0.110*** | -0.163*** | -0.172 | -0.0585 | 0.0878 | 0.305* | -0.217 |
| No Schooling (Reference) |  |  |  |  |  |  |  |
| Primary School | -0.0427 | -0.0585 | 0.112 | -0.0350 | 0.450** | -0.305 | -0.0101 |
| Secondary School | -0.111 | -0.140 | 0.162 | -0.0472 | 0.286* | -0.278 | 0.0828 |
| College | -0.117 | -0.0557 | -0.294* | -0.188 | 0.690*** | -0.398* | 0.141 |
| Post-College | -0.195 |  | -0.425*** |  |  |  |  |
| WQ1 |  |  |  |  |  |  |  |
| WQ 2 | -0.00169 | 0.0208 | 0.0872 | -0.0329 | 0.00250 | 0.137 | 0.186 |
| WQ 3 | -0.140* | -0.172* | -0.191 | -0.107 | -0.146 | -0.134 | 0.273 |
| WQ 4 | -0.0288 | -0.0780 | -0.151 | 0.0795 | -0.434** | 0.341 | 0.218 |
| WQ 5 | -0.0400 | -0.0468 | -0.410** | 0.102 | -0.0310 | -0.564 | 0.147 |
| Constant | 0.887*** | 0.904*** | 0.491 | 0.623*** | -0.403 | 0.693 | 0.335 |
| N | 3226 | 1548 | 187 | 895 | 183 | 95 | 318 |
|  | * p<0.05, ** p<0.01, *** p<0.001 | | | | | | | |

|  | **Table S23: Probability of five of more visits to catastrophic health expenditure (CHE)** | | | | | | |
| --- | --- | --- | --- | --- | --- | --- | --- |
|  | (1)  All | (2)  China | (3)  Ghana | (4)  India | (5) Mexico | (6)  Russia | (7)  South Africa |
| CD CHE case (Reference) |  |  |  |  |  |  |  |
| NCD CHE case | 0.131*** | 0.0780 | 0.210** | 0.180** | -0.0200 | -0.249 | 0.196 |
| Injury CHE case | -0.0963 | -0.204*** | 0.204 | 0.0848 | 0 | -0.305 | -0.317 |
| Other CHE case | 0.0263 | -0.0561 | 0.0143 | 0.104 | 0.0803 | -0.129 | 0.212 |
| China (Reference) |  |  |  |  |  |  |  |
| Ghana | -0.0533 |  |  |  |  |  |  |
| India | 0.147*** |  |  |  |  |  |  |
| Mexico | 0.107 |  |  |  |  |  |  |
| Russia | 0.121 |  |  |  |  |  |  |
| South Africa | 0.189* |  |  |  |  |  |  |
| Age | -0.000017 | 0.000494 | 0.00172 | -0.00068 | 0.00349 | 0.00612 | -0.00141 |
| Urban residence | -0.0612 | -0.00398 | 0.00817 | -0.119 | -0.428*** | -0.508* | -0.0145 |
| Female | 0.0492 | 0.0868* | -0.0152 | 0.0115 | -0.0374 | 0.0329 | 0.0585 |
| No Schooling (Reference) |  |  |  |  |  |  |  |
| Primary School | 0.0381 | 0.0497 | -0.0257 | 0.0365 | -0.293* | 0.136 | 0.226 |
| Secondary School | 0.102 | 0.137 | -0.126 | 0.0202 | 0.105 | 0.160 | 0.0500 |
| College | 0.110 | 0.0762 | 0.0997 | 0.174 | -0.121 | 0.229 | 0.0572 |
| Post-College | -0.234*** |  | -0.266*** |  |  |  |  |
| WQ1 |  |  |  |  |  |  |  |
| WQ 2 | -0.00711 | -0.0272 | -0.0911 | 0.0190 | 0.238 | 0.146 | 0.0412 |
| WQ 3 | 0.157*** | 0.199*** | -0.0268 | 0.114 | 0.448*** | -0.00497 | -0.186 |
| WQ 4 | 0.0979 | 0.148* | 0.0677 | -0.0141 | 0.303 | 0.0247 | -0.0763 |
| WQ 5 | 0.0844 | 0.0994 | 0.0712 | -0.0745 | 0.0928 | 0.686* | 0.0392 |
| Constant | 0.0137 | -0.00815 | -0.0372 | 0.182 | 0.227 | 0.120 | 0.280 |
| N | 3226 | 1548 | 187 | 895 | 183 | 95 | 318 |
|  | * p<0.05, ** p<0.01, *** p<0.001 | | | | | | |

**Table S.24: Out-of-pocket costs by public and private facility use**

|  | **Median OOP Public Inpatient spending**  **(USD)** | **Median OOP Private Inpatient spending**  **(USD)** | **Median OOP Public Outpatient spending**  **(USD)** | **Median OOP Private Outpatient spending**  **(USD)** | **Median NCD OOP Public Inpatient spending**  **(USD)** | **Median NCD OOP Private Inpatient spending**  **(USD)** | **Median NCD OOP Public Outpatient spending**  **(USD)** | **Median NCD OOP Private Outpatient spending**  **(USD)** |
| --- | --- | --- | --- | --- | --- | --- | --- | --- |
| China | 809 | 566 | 21 | 11 | 883 | 343 | 35 | 19 |
| Ghana | 60 | 91 | 9 | 27 | 18 | 91 | 17 | 29 |
| India | 114 | 397 | 9 | 18 | 350 | 492 | 11 | 30 |
| Mexico | 376 | 365 | 10 | 45 | - | 1,426 | 4 | 71 |
| Russia | 17 | 1,386 | 12 | 68 | 49 | 1,117 | 6 | 68 |
| South Africa | 29 | 1,069 | 1 | 40 | 34 | 917 | 6 | 51 |

Notes: USD: purchasing power parity 2017 international dollars. NCD: non-communicable disease. CD: Communicable diseases. OOP: out-of-pocket.

**Table S.25: Private sector share by disease area and location of care**

|  | **Private share of NCD inpatient visits** | **Private share of CD inpatient visits** | **Private share of NCD outpatient visits** | **Private share of CD outpatient visits** |
| --- | --- | --- | --- | --- |
| China | 3% | 6% | 21% | 36% |
| Ghana | 41% | 65% | 17% | 63% |
| India | 68% | 6% | 68% | 42% |
| Mexico | 12% | 0% | 30% | 3% |
| Russia | 2% | 35% | 18% | 25% |
| South Africa | 10% | 34% | 19% | 20% |

Notes: NCD: non-communicable disease. CD: Communicable diseases.

1. Description of random forests was based predominately on: James G, Witten D, Hastie T and Tibshirani T. 2015. *An Introduction to Statistical Learning: with Applications in R.* Springer: New York, NY. [↑](#footnote-ref-1)
2. References

   . Caruana R, Niculescu-Mizil A. 2006. An Empirical Comparison of Supervised Learning

   Algorithms. Proceedings of the 23rd International Conference on Machine Learning; Pittsburgh, PA. Available at: http://www.cs.cornell.edu/~caruana/ctp/ct.papers/caruana.icml06.pdf (Accessed June 7, 2017). [↑](#endnote-ref-1)
3. . Breiman L, Cutler A, Liaw A, Weiner W. Package ‘randomForest’. CRAN. Available at:

   https://cran.r-project.org/web/packages/randomForest/randomForest.pdf (Accessed June 7, 2017). [↑](#endnote-ref-2)
4. . Wang Q, Fu AZ, Brenner S, Kalmus O, Banda HT, et al. 2015. Out-of-Pocket Expenditure on

   Chronic Non-Communicable Diseases in Sub-Saharan Africa: The Case of Rural Malawi. *PLOS ONE* 10(1). [↑](#endnote-ref-3)
5. . Deb P, Munkin MK, Trivedi PK, 2006. Bayesian Analysis of the Two-Part Model with

   Endogeneity. Application to Health Care Expenditure. J Appl Econom 21: 1081–1099. [↑](#endnote-ref-4)
6. .  Belotti F, Deb P, Manning WG, Norton EC. 2012. tpm: Estimating Two-part Models. Stata J vv: 1–13. [↑](#endnote-ref-5)
7. . Xu K, Evans DB, Carrin G, Aguilar-Rivera AM, Musgrove P, Evans T. 2007. Protecting households from catastrophic health spending. *Health Affairs.* 26(4):972-83. [↑](#endnote-ref-6)
